# Identification of *ITPR1* gene as a novel target for hsa-miR-34b-5p in non-obstructive azoospermia: a Ca^2+^/apoptosis pathway cross-talk

**DOI:** 10.1038/s41598-023-49155-5

**Published:** 2023-12-10

**Authors:** Bahareh Maleki, Parastoo Modarres, Peyman Salehi, Sadeq Vallian

**Affiliations:** 1 Department of Cell and Molecular Biology and Microbiology, Faculty of Biological Science and Technology, University of Isfahan, Isfahan, Islamic Republic of Iran; 2 Department of Infertility, Milad Hospital, Isfahan University of Medical Sciences, Isfahan, Islamic Republic of Iran

**Keywords:** Bioinformatics, Gene expression analysis, Genetics, Molecular biology, Endocrinology, Molecular medicine

## Abstract

MiR-34b-5p has been reported as a non-invasive diagnostic biomarker for infertility. However, no gene targets regulating the mechanism of cation of this miRNA are known. In this study, using gene set enrichment analysis the Inositol 1,4,5-Trisphosphate Receptor Type 1 (*ITPR1)* gene was identified as the sole target for hsa-miR-34b-5p, and found significantly overexpressed in non-obstructive azoospermia (NOA) patients. This finding was confirmed by qRT-PCR on fresh testicular tissues from NOA patients. Then, pathway enrichment analysis as well as the diagnostic value analysis of hsa-miR-34b-5p/*ITPR1* indicated *ITPR1* as a hub gene in the calcium (Ca^2+^)-apoptosis pathway, and a valuable predictive biomarker for NOA. Moreover, gene expression and histological assays showed the association of the effects of ITPR1’s increased expression on spermatogenesis failure through induction of apoptosis in NOA patients. These data suggested that the hsa-miR-34b-5p/ITPR1 axis could serve as a potential regulatory predictive biomarker for human spermatogenesis through the Ca^2+^-apoptosis pathway cross-talk.

## Introduction

Over 20% of infertile men were diagnosed with azoospermia^1^ which is driven by blockage of the excurrent ductal system, obstructive azoospermia (OA), or by abnormal/absent testes, non-obstructive azoospermia (NOA)^2,3^. Azoospermia can occur due to defects in spermatogenesis^4^. It has been reported that microRNAs (miRNAs) were involved in the spermatogenesis process through the regulation of gene expression patterns. Therefore, the miRNA/mRNA axis has been considered an effective functional marker in various biological processes including spermatogenesis^5–8^. In particular, several studies have indicated that the miR-34b family has a crucial role as a regulatory factor on genes involved in spermatogenesis^9–11^. This family of miRNAs is highly conserved among species that involve different biological procedures including differentiation, growth, cell cycle, spermatogenesis, and apoptosis. Therefore, dysregulation of the miR-34 family could function as a regulatory effect on male infertility^12–14^. Specifically, the-miR-34b-5p has been reported as a non-invasive diagnostic biomarker for infertility^15,16^. However, the molecular mechanisms involved in miRNA/mRNA axes during human spermatogenesis are not still fully understood.

Several lines of evidence suggest that the calcium (Ca^2+^) signaling pathway is crucial for human spermatogenesis and fertility^17–20^. This secondary messenger moves across the endoplasmic reticulum (ER) membrane through localized inositol trisphosphate receptors (IP3Rs)^21,22^. Therefore, these receptors mediate different cellular processes including cell cycle, apoptosis, cell proliferation, spermatogenesis, and fertilization^23–25^. Three types of IP3R have been discovered including ITPR1-3, which are involved in Ca^2+^ influx from ER to cytoplasm in spermatogenic cells in the testes^26–29^. The different distribution of Ca^2+^ levels and the expression levels of its regulatory factors including genes and non-coding RNAs during spermatogenesis indicate the importance of the Ca^2+^ signaling pathway in balancing spermatogenic cell growth and apoptosis^26,30,31^. In other words, the potent collaboration between gene expression patterns and non-coding RNA can regulate the Ca^2+^-activated processes between the growth and survival of spermatogenic cells and cell death. Current discoveries have offered abundant evidence for crosstalk between Ca^2+^ signaling and apoptosis, but yet little is known about the mechanism involved in human spermatogenesis^17–20^.

In the present study, we aim to explore the miRNA/mRNA axis in patients with OA and NOA. In this regard, in the the present study in the first step, the expression level of hsa-miR-34b-5p was investigated in patients with NOA and OA. Then the mRNAs which were a specific target for hsa-miR-34b-5p were screened in microarray datasets from OA and NOA patients for differentially expressed genes (DEGs). Among DEGs, the *ITPR1* gene was identified as the sole target for hsa-miR-34b-5p, which was up-regulated in NOA patients.

## Methods

### Microarray datasets analysis

Integrated microarray data were used to analyze NOA and OA patients’ differentially expressed genes (DEGs). The transcription profiles from testis biopsy specimens of individuals with azoospermia were obtained from National Institue of Health-Gene Expression Omnibus (NCBI-GEO) databases (https://www.ncbi.nlm.nih.gov/geo/). Five microarray datasets were retrieved from the GEO database: (the Genomic Spatial Event, GSE), GSE108886, GSE45887, GSE45885, GSE145467, and GSE9210. After further investigation, GSE45887 was excluded because of the common samples shared between datasets GSE45887 and GSE45885. Thus, four GEO datasets were included in the analysis: (i) GSE108886 (including 8 NOA, 3 OA, and 1 pooled control testicular RNA samples), (ii) GSE145467 (including 10 OA and 10 NOA samples), (iii) GSE9210 (including 47 NOA and 11 OA patients) and (iv) GSE45885 (including 27 samples from patients with NOA and 4 with normal spermatogenesis). The datasets analysed during the current study are available in the NCBI-GEO repository (https://www.ncbi.nlm.nih.gov/geo/). More detailed information including the accession number and platforms for each dataset is included in Table 1.

**Table 1:** Detailed information on the gene expression omnibus (GEO) microarray profiles of azoospermia patients.

GEO accession no.	Type	Tissue	Samples	Selected samples		Platform	Annotation platform
			Total	NOA	OA		
GSE108886	mRNA	Testis	12	8	3	GPL10558	Illumina HumanHT-12 V4.0 expression beadchip
GSE45885	mRNA	Testis	31	14	–	GPL6244	Affymetrix Human Gene 1.0 ST Array [HuGene-1_0-st]
GSE145467	mRNA	Testis	20	10	9	GPL4133	Agilent-014850 Whole Human Genome Microarray 4 × 44K G4112F
GSE9210	mRNA	Testis	58	47	9	GPL887	Agilent-012097 Human 1A Microarray (V2) G4110B
			121	79	21		

*OA* obstructive azoospermia, *NOA* non-obstructive azoospermia, *GPL* GEO platform, *GSE* GEO series dataset.

A quantile normalization procedure in the limma package was applied to normalize the data. Relative log expression (RLE) and principal component analysis (PCA) plots were applied to evaluate the quality of the samples and to visualize unwanted variations before/after implementing the normalization. Following quality control and removing arrays with poor quality, datasets were integrated for analysis of the DEGs. To adjust batch effects among different datasets, an empirical Bayes method (Combat) was applied through the surrogate variables (sva) package. The normality of the statistical data after removing interbatch differences were visualized using quantile–quantile (Q–Q plot) and histogram plots. The DEGs between NOA and OA samples were determined by the t-statistics approach provided in the limma. P-value < 0.01 and |logFC| > 1 were used as DEG screening criteria. The P-values were adjusted using the Benjamini–Hochberg false-discovery rate method^32,33^.

### Gene/pathway enrichment and receiver operating characteristic (ROC) curve analysis

Among DEGs, the putative target(s) for hsa-miR-34b-5p were predicted via the miRWalk V2.0 database. Furthermore, Gene ontology (GO) and Kyoto Encyclopedia of Genes and Genomes (KEGG) pathway (https://www.genome.jp/kegg) enrichment analyses were conducted to highlight how the miRNA/mRNA could be driving the development of NOA. A conducted bioinformatic analysis between hsa-miR-34b-5p and microarray results was employed using the miRwalk database.

Multiple ROC curve comparisons were constructed using the easyROC web-tool-based R language environment (http://www.biosoft.hacettepe.edu.tr/easyROC/). The area under the curve (AUC) was computed to evaluate corresponding miRNA/mRNA as potential regulatory biomarkers.

### Tissue collection and processing

The study population consisted of 63 fresh testicular tissue samples from infertile men who had undergone microsurgical testicular sperm extraction (micro-TESE) surgery. All samples were collected from the infertility ward of Milad Hospital of Isfahan and Isfahan Ordibehesht surgery center (Isfahan, IR Iran) between November 2018 and February 2020. All patients underwent semen testing based on the world health organization (WHO) fifth edition guidelines (2010) and the total specimens/patients were clinically and histopathologically diagnosed as azoospermia. All samples were immersed in RNA later™ Solution (Qiagen, Germany, Cat. no: 160013558) overnight at 4 °C, and then stored at − 80 °C for further processing. To minimize any possible bias, blinding, and randomization were implemented in all stages of the experiments, including sample collection, processing, and analysis. The tissue samples of patients were collected in compliance with the ethical protocol and standards of the infertility ward of Milad hospital of Isfahan and Isfahan Ordibehesht surgery center clinic.

The study population was classified into two groups conditions: (i) 45 NOA testicular samples with impaired maturity in the spermatogenic cells in the case group, (ii) 18 OA testicular samples with normal spermatogenesis in the control one. Patients were with a mean age of 29.1 ± 3.65 years (range 21–44 years) and all of them in both groups were of Iranian-Persian ethnicity. In the NOA group, the mean patient age was 31.809 ± 5.706 years (range 20–59 years); in the OA group, the mean patient age was 28.333 ± 4.802 (range 27–48). Table 2 represents the main clinical parameters of the patients.

**Table 2:** The Clinical demographic profile of the patients used in the current study.

Variable	MRR	NOA (n = 45)	OA (n = 18)
Age (years)	–	31.809 ± 5.706	28.333 ± 4.802
FSH (IU/mL)	1.4–18.1	19.366 ± 5.943	8.255 ± 4.099
LH (IU/mL)	1.24–7.8	6.045 ± 2.685	5.666 ± 2.774
TT (nmol/L)	*8.64–29*	8.809 ± 3.203	12.024 ± 4.640
Vit.D (ng/mL)	*20–40*	11. 731 ± 014	18.600 ± 3.807
Ca(mg/dL)	8.6–10.3	13.270 ± 024	9.217 ± 102

The data were represented as Mean ± SD.

*OA* obstructive azoospermia, *NOA* non-obstructive azoospermia, *MRR* male reference range, *FSH* follicle-stimulating hormone, *LH* luteinizing hormone, *TT* total testosterone, *Vit.D* vitamin D, *Ca* calcium.

Significant values are in italic.

### Total RNA extraction (including miRNA), complementary DNA (cDNA) synthesis, and gene expression experiments

According to the manufacturer’s recommendations, total RNA was isolated from testicular tissue samples using the Maxzol reagent (MaxCell, IR Iran). The purity and integrity of total RNA were evaluated using a spectrophotometer (NanoDrop OneC, Thermo Fisher Scientific, USA), and 1.5% denaturing agarose gel electrophoresis, respectively (Supplementary Fig. S2). cDNA synthesis was performed using BONmiR High Sensitivity miRNA 1st Strand cDNA Synthesis kit (Stem Cell Technology, Tehran, Iran), and easy™ cDNA synthesis kit (Parstous, Tehran, Iran) for miRNAs and gene, respectively. The experiments were carried out according to the manufacturer’s protocols.

The primers used for gene expression experiments were designed by Allele ID primer design software version 7.5 (Premier Biosoft, USA) and Oligo 7 primer analysis software version 7.6 (Molecular Biology Insights, USA). Then, the NCBI-primer BLAST service (https://www.ncbi.nlm.nih.gov/tools/primer-blast/) was applied to investigate sequence identity in the sequence databases. All primers were synthesized by Metabion, Germany. The primers were presented in Supplementary Table S1.

The qRT-PCR reactions were performed in triplicates in a Chromo 4 System qRT-PCR detector (Bio-Rad, USA) according to standard procedures. Results were expressed as cycle threshold (Ct) values. Finally, PCR products of the target genes and miRNA were loaded on 2% and 4% agarose gel, respectively (Supplementary Fig. S3). Relative gene expression analysis was performed using the comparative Ct method (2^−ΔΔCt^). The optimized conditions for qRT-PCR are shown in Supplementary Table S2.

### Histological apoptosis assays

To investigate the process of apoptosis in testicular tissues and quantify the apoptotic cells, a segment of testicular tissues from each group (NOA and OA) with a size of 2 × 2 × 2 mm was fixed in 10% formalin and paraffin-embedded. Then, 5-μm-thick cross-sections were prepared for subsequent hematoxylin–eosin (H&E) staining and deoxy-UTP-digoxigenin nick end labeling (TUNEL) assay (Promega kit, USA). To evaluate the staining intensity of processed slides, all the immunostaining data were standardized and quantified by Akiron® NEO device (Germany).

### Hormone assays

Serum follicle-stimulating hormone (FSH), luteinizing hormone (LH), total testosterone (TT), Vitamin D (Vit.D), thyroid-stimulating hormone (TSH), and Ca^2+^ levels in the patient's peripheral blood were measured using standard laboratory kits (Bio-Idea Co. Tehran, Iran).

### Statistical analysis

The sample power was calculated using the G*power Software (V3.1.9.6, Germany). A power analysis for an independent t-test indicated that the minimum sample size to yield a statistical power of at least 0.80 with an alpha of 0.05 and a large effect size (d = 0.8) is 58. Mean expression levels of the hsa-miR-34b-5p and *ITPR1* gene were computed between NOA and OA groups using the independent T-Test. Correlations between miRNA/mRNA expression levels and demographic characteristic parameters were assessed by the Pearson correlation coefficient. All analyses were performed in IBM SPSS V22.0 (IBM SPSS Inc., Chicago, USA) and GraphPad Prism 8 (GraphPad Software V8.4.2, LLC).

### Ethics declarations

This study was approved by the ethics committee of the University of Isfahan (Approval ID: IR.UI.REC.1398.090), and all experiments were performed in accordance with relevant guidelines and regulations.

### Consent to participate

Written informed consent was obtained from each patient.

## Results

### Identification of *ITPR1* gene as an hsa-miR-34b-5p target in NOA

**Figure 1:**
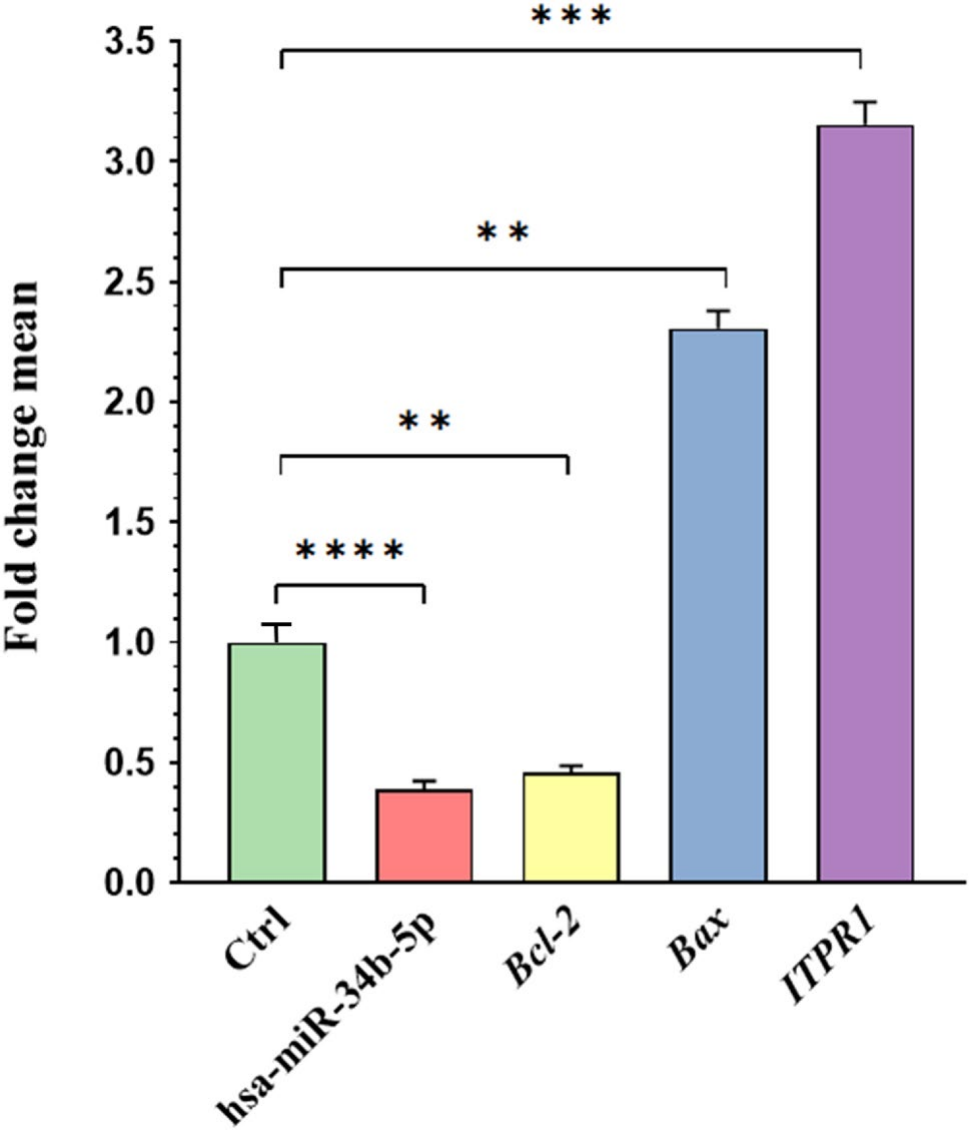
The relative expression level of hsa-miR-34b-5p, *Bcl-2*, *Bax,* and *ITPR1* in NOA and OA patients. The expression of hsa-miR-34b-5p, *Bcl-2*, *Bax,* and *ITPR1* was investigated in NOA (n = 45) compared to OA (n = 18) patients using qRT-PCR. The expression results were shown as fold change mean, considering OA samples as control (Ctrl) for all the genes tested. The data were depicted as the standard error of the mean with SEM. **P < 0.01, ***P < 0.001, and ****P < 0.0001.

The relative expression levels of hsa-miR-34b-5p in patients with non-obstructive azoospermia (NOA) and obstructive azoospermia (OA) were investigated. As presented in Fig. 1 and Table 3, the data showed significant down-regulation of hsa-miR-34b-5p expression in the NOA as compared to OA patients (FC = 0.387, P-value < 0.0001).

**Table 3:** Evaluation of genes expression levels in non-obstructive azoospermia (NOA) compared to obstructive azoospermia (OA) groups.

Marker	P value	Interpretation	Log_2_ fold change	Fold change	Significantly different
Hsa-miR-34b-5p	< 0.0001	2.58-fold down-regulated	− 1.369	0.387	YES****
*ITPR1*	< 0.001	3.15-fold up-regulated	1.656	3.152	YES***
*Bcl-2*	< 0.01	2.16-fold down-regulated	− 1.117	0.461	YES**
*Bax*	< 0.01	2.30-fold up-regulated	1.206	2.307	YES**

**P < 0.01, ***P < 0.001 and ****P < 0.0001.

**Figure 2:**
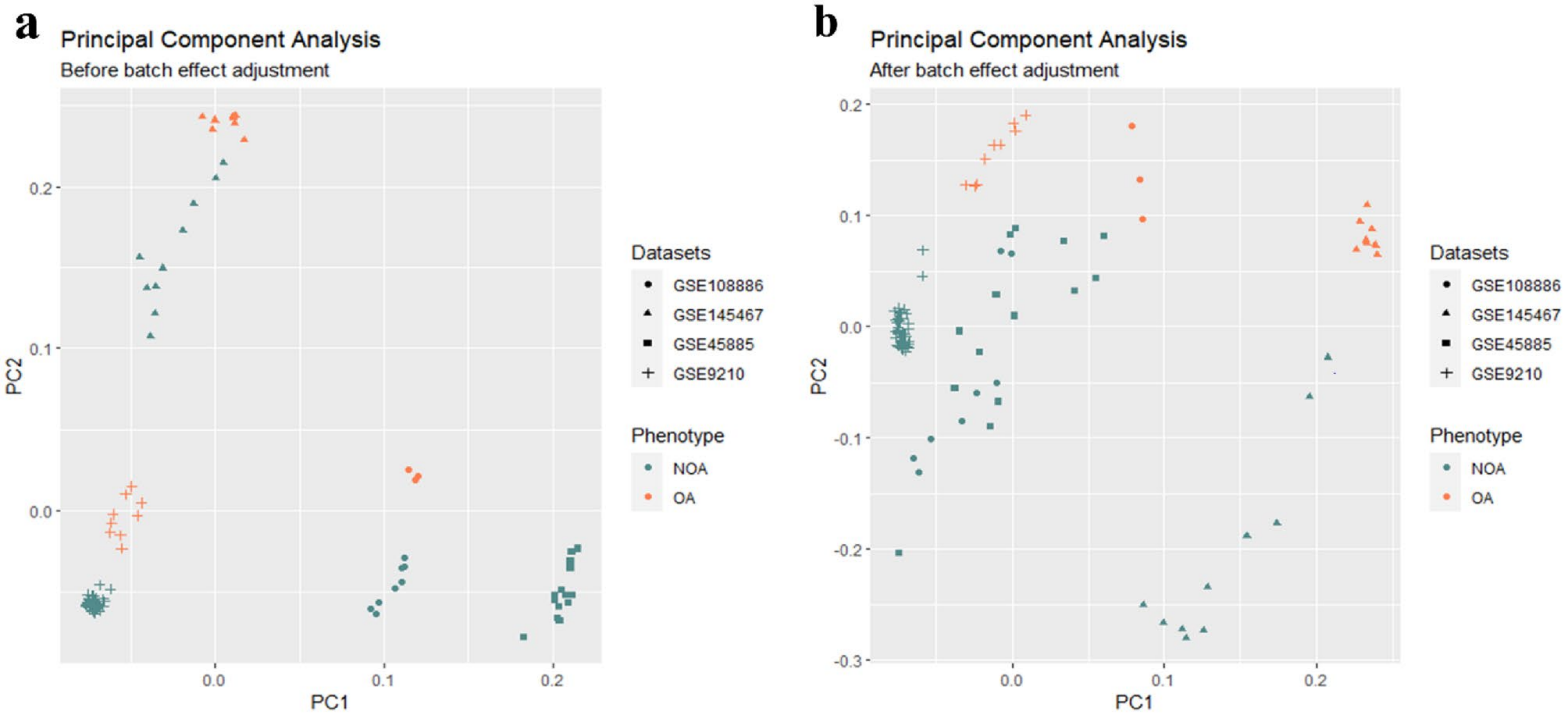
Principal component analysis (PCA) plot. PCA plots illustrate the clustering pattern of normalized samples before (**a**) and after (**b**) batch effect adjustment. The datasets and NOA and OA samples were shown.

Next, to explore the target genes (mRNAs) associated with hsa-miR-34b-5p in NOA patients, microarray RNA datasets from 92 NOA and 24 OA testicular tissue samples were analyzed for candidate RNAs (see Table 3 for more details of the microarray RNA datasets). After conducting normalization, quality control, and removing arrays of poor quality, a total of 100 samples (including 79 NOA and 21 OA) were finally obtained to be integrated for analysis of the DEGs (Supplementary Table S3). Figure 2 shows PCA plots that visualize the sample clustering patterns with or without batch effect adjustment. Furthermore, the histogram provided a visual representation of the data distribution, and the Q-Q plot was used to compare our data distribution with a theoretical normal distribution. Both plots suggested that our data followed a normal distribution (Supplementary Fig. S1).

**Figure 3:**
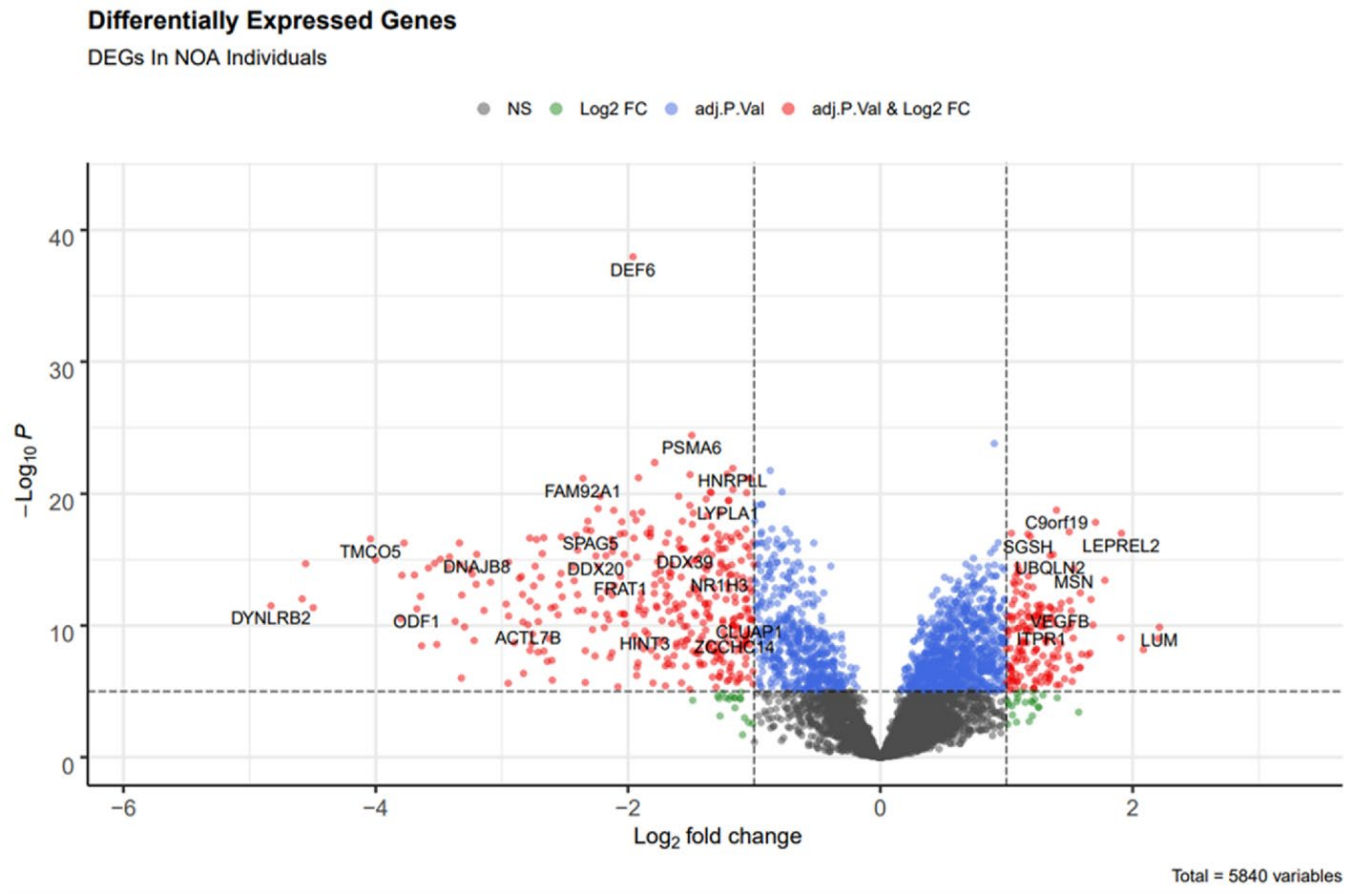
Volcano plot of differentially expressed genes (DEGs). The cut-off for screening the statistical significance DEGs was considered P-value < 0.01, adj.P-value < e−10 and |logFC| > 1. The red dot points present more significant genes such as the *ITPR1* gene with logFC: 1.3, P-value: 3.37E−12, and adj. P-value: 3.12E−11 as statistical significance.

The data showed that 350 genes were differentially expressed between NOA and OA groups. These included 80 up-regulated and 270 down-regulated genes. They were identified using to fit a linear model and applying the t-statistics test provided in the limma package. P-values < 0.01 and |logFC| > 1 were considered the threshold for screening the DEGs. Figure 3 represents the volcano plot of DEGs in NOA patients compared to OA ones. The list of DEGs associated with the occurrence of OA is presented in Supplementary Table S4.

### Involvement of *ITPR1* in the hsa-miR-34b-5p regulatory pathway

Gene set enrichment analysis on DEGs and hsa-miR-34b-5p target genes suggested that *ITPR1* (logFC: 1.336552, P-value: 3.37e−12) may be involved in the development of NOA through the hsa-miR-34b-5p regulatory pathway. Interestingly, analysis of the miRWalk database indicated that *ITPR1* formed a miRNA/mRNA duplex with the hsa-miR-34b-5p by the highest score of 1.00. Table 4 represents a detailed miRNA/mRNA duplex based on the miRWalk database that the binding site is determined using the RNAduplex program from the ViennaRNA software package.

**Table 4:** Detailed RNAduplex information based on miRWalk database.

predicted pairing of the target region of *ITPR1* (bottom) and hsa-miR-34b-5p (top)	Score	Position	Binding site	Energy
TAGGCAGTGTCATTAGCTGATTG&GTTGGCATGATGACATTTCATTTGTGCCA..(((((((((((((((((((..&)))))).)))))))))……..)))).	1	3ʹ UTR	8812–8841	− 19.9
TAGGCAGTGTCATTAGCTGATTG&AATTATCCTCTGGTGATGCTGTTTC .(((((((((((((((..(((..&.)))..))))))))))))))).	0.92	3ʹ UTR	9161–9186	− 19.1

“.”—Denotes bases that are essentially unpaired.

“&”—Character as a separator.

“()”—Strongly (> 66%) up-/downstream paired bases.

### *ITPRI* modulates the Ca^2+^-apoptosis signaling pathway in NOA

**Figure 4:**
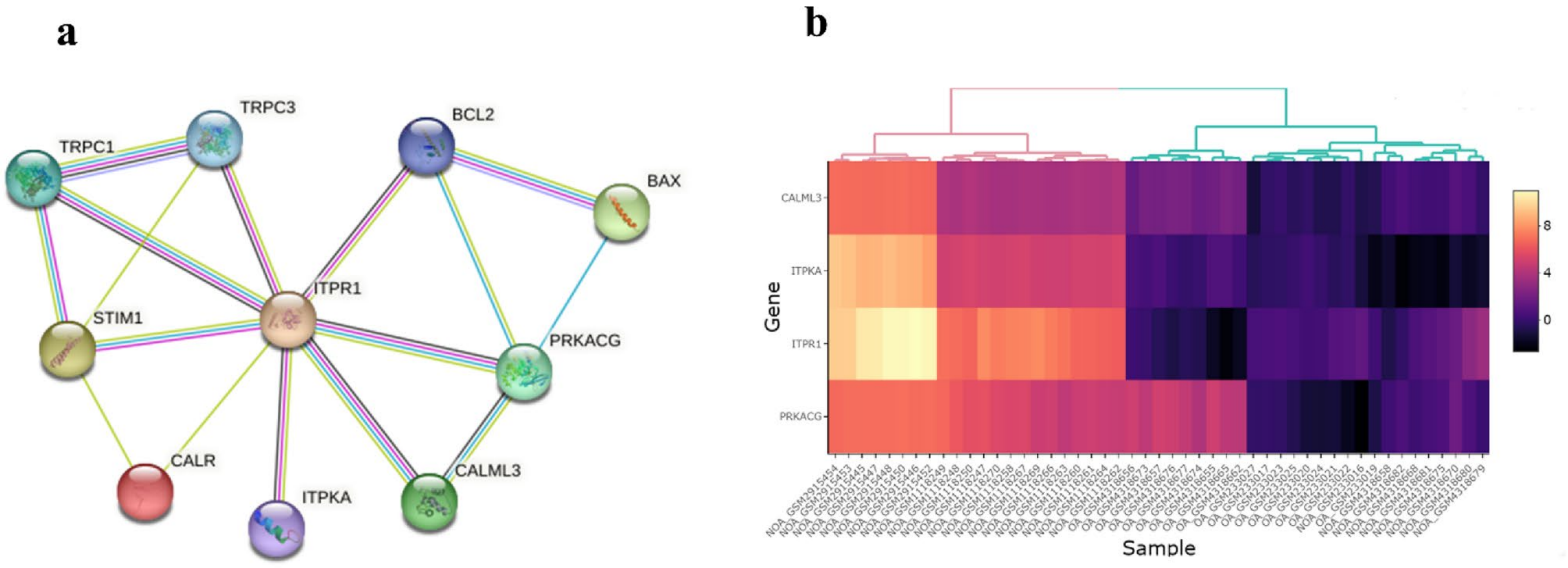
Protein–protein interaction (PPI) network and heatmap of DEGs. (**a**) Protein–protein interaction (PPI) network of azoospermia dysregulated genes associated with Ca^2+^/apoptosis signaling pathway based on KEGG biological pathways. The protein–protein association network was retrieved from the STRING enrichment web service (https://string-db.org/). (**b**) Heatmaps of four differentially expressed genes (*CALML3*, *ITPKA*, *ITPR1*, and *PRKACG*) between NOA and OA samples. Yellow and purple represent high and low expressions, respectively.

To explore the role of *ITPR1* in the molecular pathogenesis of NOA, pathway enrichment analysis was performed. The analysis of the extracted KEGG pathways suggested that the interplay of *ITPR1* and hsa-miR-34b-5p was involved in the development of NOA through Ca^2+^ and apoptosis signaling pathways. Moreover, DEGs related to the Ca^2+^ and apoptosis pathways were mapped using the search tool for retrieval of interacting genes (STRING) (https://string-db.org) database. As shown in Fig. 4a, the Protein–protein interaction (PPI) network indicated that *ITPR1* can act as a hub gene in the positive regulation of apoptosis. Furthermore, it was found that *Bcl-2* and *Bax*, as two important hallmarks proteins in the apoptosis pathway, had good connectivity with the *ITPR1* in the PPI network. The heatmaps of DEGs related to the Ca^2+^-apoptosis signaling pathway are represented in Fig. 4b.

### Significant expression of *ITPR1* in NOA patients

Given the results from the miRWalk database indicating the formation of the *ITPR* /hsa-miR-34b-5p duplex, we were prompted to investigate the expression of *ITPR1* in testicular tissue samples from patients with NOA samples. Interestingly, the results showed a significant increase in the expression of *ITPR1* (P-value < 0.001). This data indicated that our meta-analysis and experimental results were in the same direction. Figure 1 and Table 3 show the altered expression levels of *ITPR1* in NOA and OA patients.

### Altered expression of apoptosis-related genes, *Bcl-2* and *Bax,* in NOA

Given the role of *Bcl-2* and *Bax* in the Ca^2+^-apoptosis pathway and the involvement of *ITPR1* as a hub gene in the positive regulation of apoptosis, their expression levels were evaluated in NOA and OA patients. As seen in Fig. 1, the *ITPR1* showed an increased expression in NOA, and its increased expression was associated with a 2.30-fold increase in *Bax* (pro-apoptotic gene) expression and a 2.16-fold decrease in *Bcl-2* (anti-apoptotic gene) expression. All dysregulations were significant with a P-value < 0.01 (Table 3). These findings suggested that the increased expression of *ITPR1* in NOA could be associated with the induction of apoptosis in testicular tissues most likely through up-regulation of *Bax* and down-regulation of *Bcl-2* genes expression (see Fig. 1).

### Hsa-miR-34b-5p/*ITPR1* associates with the induction of apoptosis in NOA

**Figure 5:**
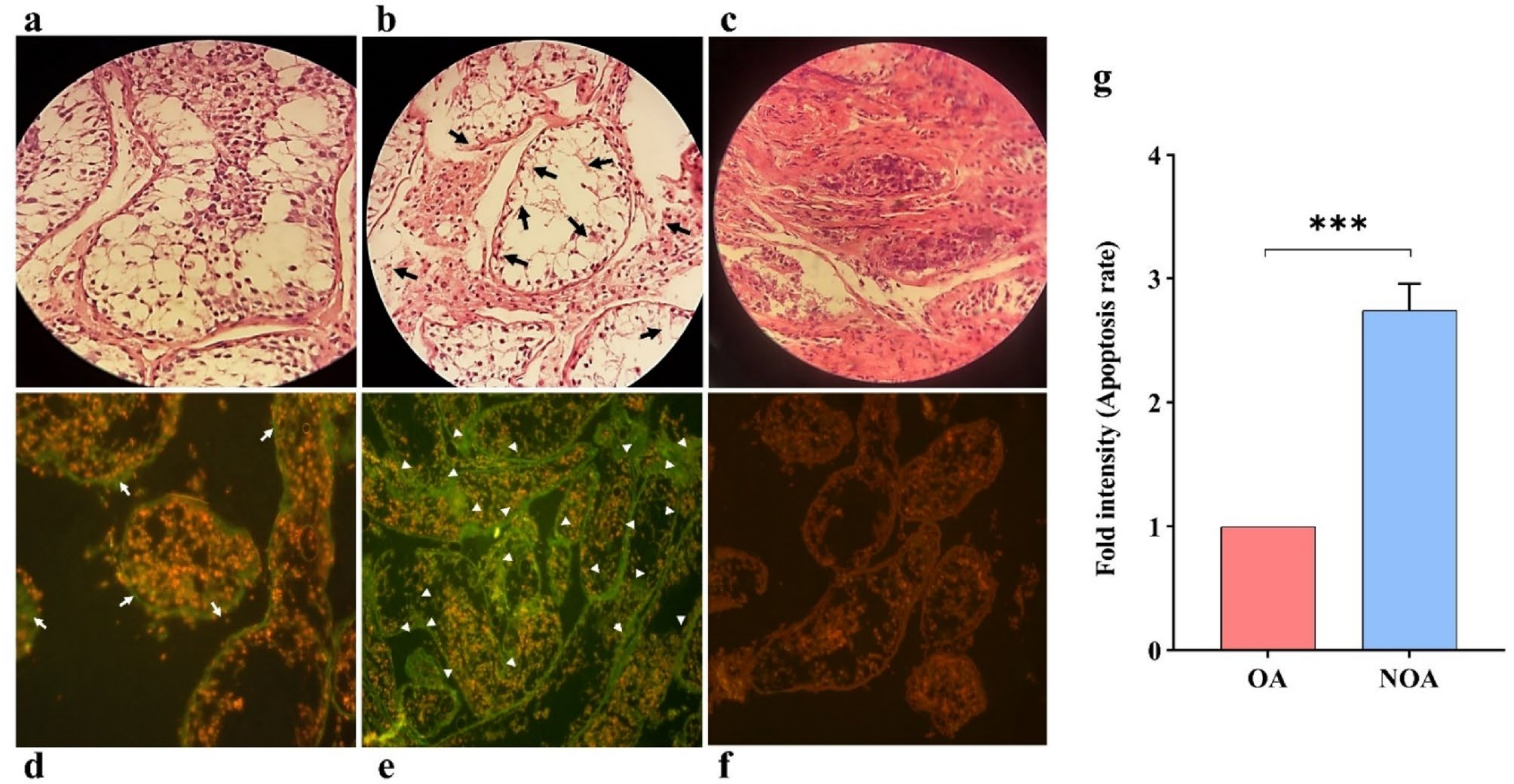
Histological feature of the human testicular tissues. (**a–c**) H&E stained testis section from adult OA and NOA. (**a**) OA testicle (Control). (**b**) NOA testicle. (**c**) NC. Magnification: × 40. *H&E* Hematoxylin and eosin, *NOA* non-obstructive azoospermia, *OA* obstructive azoospermia, *NC* negative control. Arrows: Irregularity in NOA patients’ seminiferous. (**d–f**) TUNEL-stained sections of the human testicular tissue for the detection of apoptosis. (**d**) OA, control group. A few TUNEL-stained nuclei were observed in the OA (Arrowheads). (**e**) In the NOA, the increase in the apoptotic cells was observed compared to the OA group (Arrows). (**f**) *NC* negative control. Green and Red fluorescent stained nuclei indicate apoptotic and viable cells, respectively. Magnification, × 40. *TUNEL* deoxy-UTP-digoxigenin nick end labeling, *NC* negative control. (**g**) Quantification of staining intensity reveals 2.7-fold increases in apoptosis rate in NOA compared with OA (control group).

To investigate the association of the hsa-miR-34b-5p/*ITPR1* axis with apoptosis in the testicular tissue of NOA, terminal deoxynucleotidyl transferasedUTP nick end labeling (TUNEL) assy as well as histopathological analysis were carried out. As illustrated in Fig. 5a,b hematoxylin–eosin (H&E) staining showed the presence of spermatogenic cells in OA testicular tissues with normal spermatogenesis, while the NOA testicular tissues showed impaired spermatogenesis. Moreover, as shown in Fig. 5c,d, the testes of the OA showed only a few TUNEL-positive cells. These changes were observed occasionally in the testes of OA. Whereas the testes of NOA exhibited a large number of TUNEL-positive germ cells, indicating the induction of apoptosis. Also, quantification of immunofluorescence staining intensity showed that apoptotic cells are about 2.7-fold more abundant in NOA than in OA (Fig. 5g). Overall, the results obtained from both H&E and TUNEL methods showed an increase in the frequency of apoptosis in the seminiferous epithelium of NOA as compared with OA patients. It is notable that to avoid false positive results, negative controls were included in the immunostaining analysis (Fig. 5c,f).

### *ITPR1* functions as a predictive biomarker for NOA

**Figure 6:**
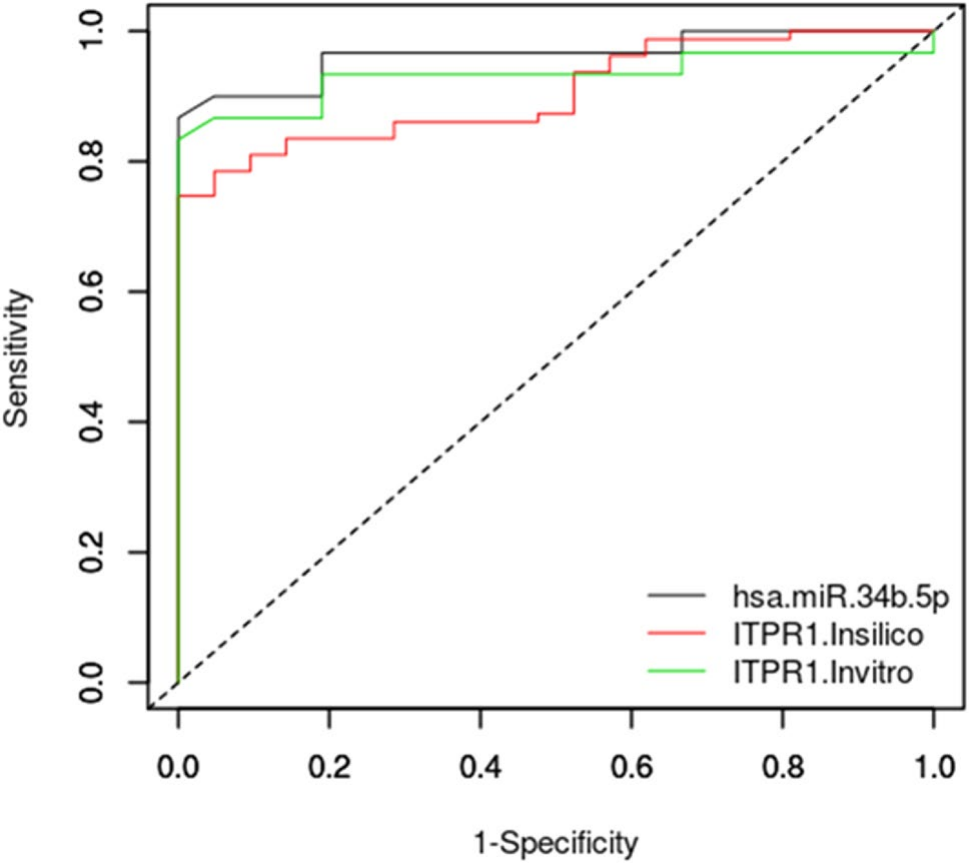
Multiple comparisons of correlated areas under the ROC curves (AUC) for DEGs and studied markers. ROC curves were performed using an easyROC web-tool-based R language environment (http://www.biosoft.hacettepe.edu.tr/easyROC/) to assess the predictive accuracy of studied markers for male infertility. The AUC values were ≥ 0.9 demonstrating the predictive power of the identified markers.

Given the significant expression of *ITPR1* gene expression in NOA compared to OA samples, a multiple-comparisons of correlated areas under the ROC curves (AUC) were performed for *ITPR1* and hsa-miR-34b-5p using easyROC. Figure 6 shows the ROC curves for both RNAs with desirable AUCs ≥ 0.90 and a significant P-value < 0.0001. Moreover, the specificity and sensitivity for both markers were more than 85%. The results revealed that *ITPR1* and has-miR-34b-5p could be valuable predictive biomarkers for NOA.

### Expression of *ITPR1* correlates with increased serum Ca^2+^ in NOA patients

Because of the known involvement of *ITPR1* in cellular calcium influx and its association with the progression of infertility in NOA patients, the level of Ca^2+^ as well as other clinicopathological factors related to infertility including FSH, LH, TT, and Vit. D were investigated. The results were analyzed by applying the fold change value of the Bivariate Correlations test (see Table 5). Interestingly, the valuation of blood Ca^2+^ level showed a strong correlation between this variable and the expression level of markers with a P-value < 0.01. The correlation between the expression level of both markers (hsa-miR-34b-5p, *ITPR1*) and age was meaningful (P < 0.05). Confirming the significance level of the FSH and LH variables with P < 0.05 can be attributed to the strong association between these factors and the expression levels of the studied markers. Unlike hsa-miR-34b-5p, increased expression of *ITPR1* was negatively correlated with Vit. D variable. TT level demonstrated no influential relevance.

**Table 5:** Correlations between expression level of studied markers and clinical characteristic parameters.

Marker	Pearson correlation coefficients (PCC)					
	Age	FSH	LH	TT	Vit.D	Ca
hsa-miR-34b-5p	− 0.640*	− 0.624**	− 0.301*	0.285^ns^	− 0.148^ns^	− 0.676*
*ITPR1*	0.693*	0.641**	0.589**	− 0.192^ns^	− 0.599*	0.792**

*FSH* follicle-stimulating hormone, *LH* luteinizing hormone, *TT* total testosterone, *Vit.D* vitamin D, *Ca* calcium, *ns* non-significant.

*P < 0.05, **P < 0.01.

## Discussion

MicroRNAs play an important regulatory role in gene silencing at the post-transcriptional level^5–8^, however, their impact on human spermatogenesis is not fully understood^11^. Among these molecules, the involvement of the miR-34 family in spermatogenesis has been the focus of recent attention^34–36^. This family includes hsa-miR-34a, -miR-34b, and -miR-34c which potentially play a crucial role in the control of the cell cycle and apoptosis^37,38^. Several reports indicated the down-regulation of hsa-miR-34b as a probable cause of the occurrence of male infertility^3,11,39^. Moreover, increased expression of hsa-miR-34b has been reported in the adults’ testes in contrast to the pre-pubertal, implying its importance in spermatogenesis^40^. Furthermore, evaluation of differential miRNA expression of NOA and normal testicular tissues, suggested reduced levels of hsa-miR-34b-5p as a predictive biomarker for NOA^11,41^. In the present study, we first evaluated the expression of hsa-miR-34b-5p in human testicular tissues and found a significant decrease in the expression of hsa-miR-34b-5p in NOA patients compared to obstructive OA. Next, to study the hsa-miR-34b-5p-related pathogenesis of NOA in detail and to find possible miRNA/mRNA axis, we performed a meta-analysis on microarray data from testicular tissues of NOA and OA patients. The data resulted in the identification of 80 DEGs which were highly expressed in the NOA. Among these, interestingly the Inositol 1, 4, 5-trisphosphate receptor type 1 (*ITPR1)* gene was predicted as the only target of hsa-miR-34b-5p with high scores. QRT-PCR analysis confirmed the high level of *ITPR1* expression in NOA as compared to OA tissues. Therefore, the down-regulation of hsa-miR-34b-5p could be attributed to the increased expression of *ITPR1* as its potential target. This may indicate that the expression of *ITPR1* is negatively regulated by the hsa-miR-34b-5p in spermatogenesis. The exact mechanism of the regulation of the expression of hsa-miR-34b is not fully understood, however, alteration of the methylation pattern of hsa-miR-34b promoter^36^, as well as SUMOylation of the upstream factors including Akt (which phosphorylate FOXO3 transcription factor) has been proposed^42^.

Our prediction data as depicted from the miRWalk database indicated that the hsa-miR-34b-5p could target the *ITPR1* gene with a score of 1 (see Table 4). Therefore, it could be hypothesized that the decrease in the expression of hsa-miR-34b-5p could negatively regulate the expression of *ITPR1* in NOA testicular tissues. It has been recently reported that a gain-of-function mutation in the *ITPR1* gene could result in its hyperactivation followed by male infertility in mice^23–25^. This finding could support our finding of the association of the increased expression of *ITPR1* in male infertility in humans.

ITPR1 is an endoplasmic reticulum (ER) Ca^2+^ channel which is activated by inositol 1,4,5-trisphosphate (IP3) second messenger and modulates intracellular calcium homeostasis and signaling in the cell^25^. It has numerous pivotal roles in different cellular processes including cell proliferation, differentiation, metabolism, phagocytosis, autophagy, and immune regulation^25,43^. Evidence has shown that *ITPR1* could function as a direct novel target of hypoxia-inducible factor subunit 2 alpha (*HIF2α)* involving in the activation of autophagy. Therefore, increased expression levels of *ITPR1* by *HIF2α* regulate natural killer (NK) cells-induced autophagy. As *HIF2α* has been documented to be a required factor in tumor progression in renal cancer cells^44^, it is possible that *ITPR1* is involved in conducting tumor growth and protecting the cancer cells against NK cells by *HIF2α*^45^. This study could provide insight into the connection between *HIF2α/ITPR1* axis and NK cells in regulating renal cancer cells. In addition to the role of this gene in oncological diseases, studies have indicated that *ITPR1* was significantly overexpressed in neurodegenerative disorders such as Alzheimer compared to the control group^46,47^. This protein may impact intracellular calcium homeostasis through interaction with β-amyloid peptide and inactivation of endothelial nitric oxide synthase (eNOS) resulting in apoptosis^47,48^. Therefore, *ITPR1* could be introduced as a critical protein involved in the regulation of Ca^2+^ homeostasis and apoptosis in the pathogenesis of Alzheimer’s disease.

In addition to *ITPR1*, our gene set enrichment analysis resulted in three additional DEGs including *CALML3, ITPKA*, and *PRKACG* involved in Ca^2+^-related signaling pathways (see Fig. 4). Our data on the protein–protein interaction (PPI) network depicted a strong interaction between these genes that *ITPR1* operates as a hub gene in the Ca^2+^-associated signaling pathways. Interestingly, the role of these genes has been likewise determined in the Ca^2+^ and apoptosis signaling pathways. For instance, *CALML3* overexpression inhibits human lens epithelial (HLEB-3) cell apoptosis by activating the PI3K/Akt pathway^49^. Differentially down-regulated *ITPKA* gene is involved in inositol phosphate metabolism and subsequently, Ca^2+^ influx and apoptotic signaling pathway^50^. Also, *PRKACG* may affect neuron membrane potentiation, conducting apoptosis through activation of the MAPK-JNK signaling pathway and Ca^2+^/cAMP metabolism deregulation^51^. Although these findings show that all four genes may be involved in the cross-talk of the Ca^2+^-apoptosis signaling pathway, only *ITPR1* was targeted by hsa-miR-34b-5p.

It has been documented that sperm ion channels including Ca^2+^ play significant regulatory roles in the process of spermatogenesis, controlling sperm maturity and motility, the acrosome response, and fertilization^17,52^. These Ca^2+^ ions are accumulated in spermatogenic cells at different developmental stages and manage the spermatogenesis and spermiogenesis processes^53^. Therefore, irregular Ca^2+^ signaling pathways can impair testosterone levels, which leads to unnatural spermatogenesis and even complete male infertility Furthermore, several reports have shown that the calcium pathway could be interconnected with the apoptosis pathway^26,54–56^.

**Figure 7:**
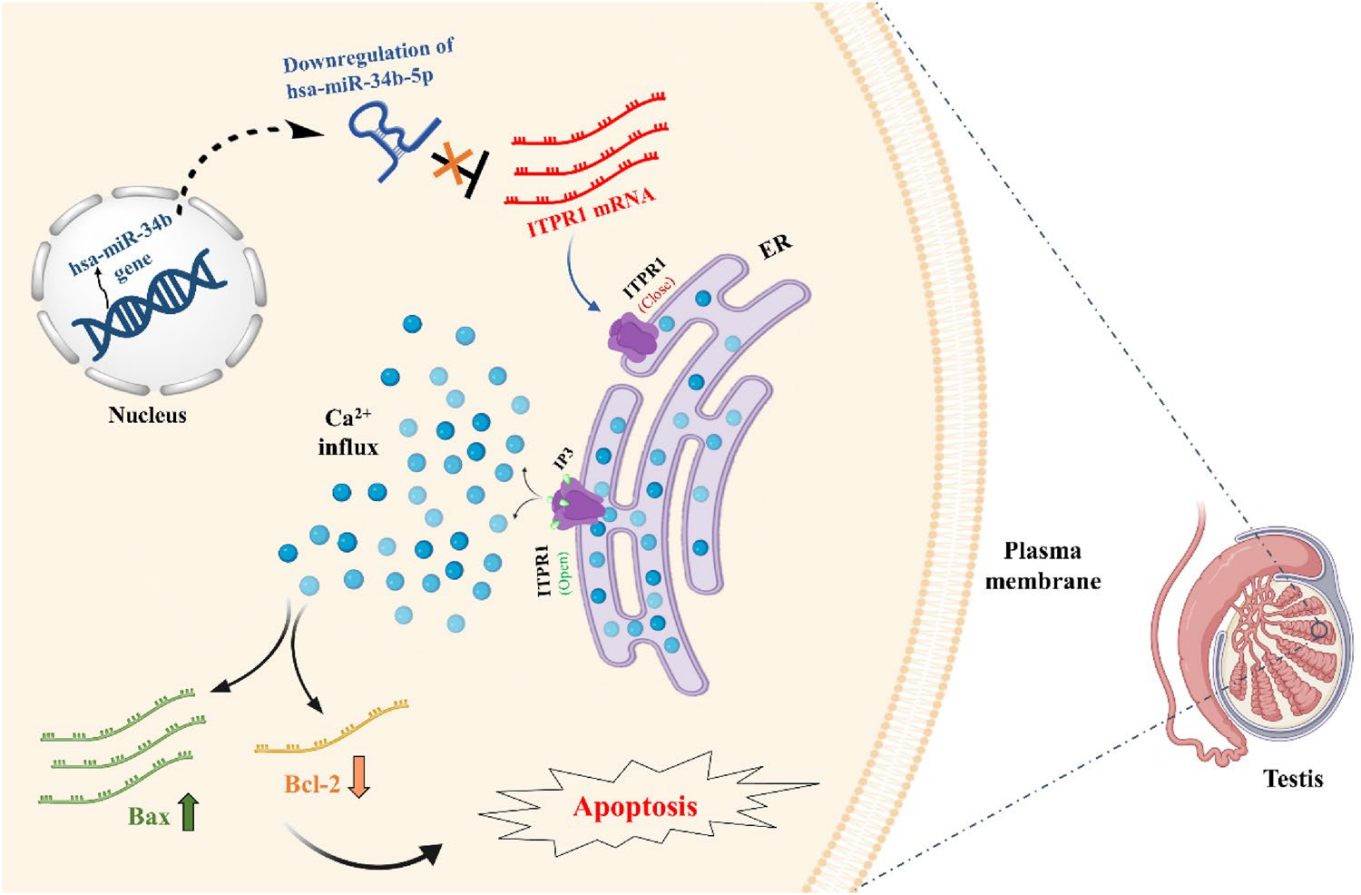
A schematic diagram for the function and mechanisms of the hsa-miR-34b-5p/*ITPR1* axis in male infertility in human. The Figure depicts hsa-miR-34b-5p acts as a silencer on 3ʹ UTR of *ITPR1* expression. Therefore, down-regulation of hsa-miR-34b-5p will follow overexpression of *ITPR1* mRNA in human testes. Subsequently, Ca^2+^ influx occurs from ER to cytoplasm in spermatogenic cells in the teste. Finally, this influx creates a cross-talk between Ca^2+^ and apoptosis pathways through unknown upstream factors.

In line with the above findings, our results showed that the high level of calcium released by the ER, possibly through a *Bax/Bcl-2*-dependent mechanism is thought to promote apoptosis in testis cells. However, low levels of release of ER calcium likely provoke cell survival, indicating that ER calcium sources are the critical regulators of cellular apoptosis. Using histological approaches including the hematoxylin–eosin (H&E) staining technique and TUNEL assay, we found a significantly high level of apoptosis in spermatogenic cells in NOAs compared to OAs (see Fig. 5). The alteration of histopathologic patterns in the testicular tissues of NOA patients may suggest a functional role for the hsa-miR-34b-5p/*ITPR1* axis in male fertility. Moreover, the positive correlation of increased expression of the *ITPR1* gene with increased levels of serum calcium in NOA patients could confirm the association of *ITPR1* gene expression with infertility through Ca^2+^ influx/apoptosis pathway crosstalk (see Fig. 7).

In summary, this study for the first time provided a link between the dysregulation of the hsa-miR-34b-5p/*ITPR1* axis and male infertility in humans. Moreover, the observed gene expression alteration showed an association with apoptosis likely through the Ca^2+^-apoptosis signaling pathway. Therefore, the hsa-miR-34b-5p/*ITPR1* axis could be introduced as a novel potential predictive biomarker in male infertility in humans.

However, our study has several limitations. The findings are primarily derived from gene expression data, and validation at the protein level has not been conducted. Techniques such as Western blotting or animal model validation could assist in confirming detailed signaling pathways and elucidating the precise biological functions of *ITPR1* gene. Despite these acknowledged limitations, it is believed that the present study makes a significant contribution to the field of human infertility and lays the groundwork for more comprehensive future research.

### Supplementary Information

Supplementary Information.

## Data Availability

All study data are included in the Supplementary Information file. The datasets analysed during the current study are available in NCBI-GEO databases (https://www.ncbi.nlm.nih.gov/geo/).
