# Author Correction: Identification of *ITPR1* gene as a novel target for hsa-miR-34b-5p in non-obstructive azoospermia: a Ca^2+^/apoptosis pathway cross-talk

**DOI:** 10.1038/s41598-024-53786-7

**Published:** 2024-02-14

**Authors:** Bahareh Maleki, Parastoo Modarres, Peyman Salehi, Sadeq Vallian

**Affiliations:** 1 Department of Cell and Molecular Biology and Microbiology, Faculty of Biological Science and Technology, University of Isfahan, Isfahan, Islamic Republic of Iran; 2 Department of Infertility, Milad Hospital, Isfahan University of Medical Sciences, Isfahan, Islamic Republic of Iran

Correction to: *Scientific Reports* 10.1038/s41598-023-49155-5, published online 10 December 2023

The original version of this Article contained an error in Reference 9, which was incorrectly given as:

Piryaei, F. *et al.* Global analysis in non-obstructive azoospermic testis identifies miRNAs critical to spermatogenesis. *Andrologia* **1**, 1 (2022).

The correct reference is listed below:

Piryaei, F. *et al.* Global analysis in nonobstructive azoospermic testis identifies miRNAs critical to spermatogenesis. *Andrologia* **2023**, Article ID 2074931 (2023). 10.1155/2023/2074931.

The original Article has been corrected.

