# Supplementary material for: Identification of ITPR1 gene as a novel target for hsa-miR-34b-5p in non-obstructive azoospermia: a Ca2+/apoptosis pathway cross-talk
Source: Sci Rep. 2023 Dec 10;13:21873. doi: 10.1038/s41598-023-49155-5 (PMC10710998; doi:10.1038/s41598-023-49155-5)
Supplement: Supplementary file 1 — Supplementary Information. [file 41598_2023_49155_MOESM1_ESM.docx]

**Supplementary information**

**Article Title:** Identification of ITPR1 gene as a novel target for hsa-miR-34b-5p in non-obstructive azoospermia: A Ca2+/apoptosis pathway cross-talk

Bahareh Maleki^1^, Parastoo Modarres^1^, Peyman Salehi^2^ and Sadeq Vallian^1*^

***Corresponding author:**

Sadeq Vallian, MSc, PhD

Prof. of human molecular genetics

University of Isfahan

Division of Genetics, Department of Cell and Molecular Biology and Microbiology, Faculty of Biological Science and Technology, University of Isfahan, Isfahan, IR Iran.

Phone: +98 31 37932456

Email: svallian@sci.ui.ac.ir

**Emails**

b.maleki@sci.ui.ac.ir

parastoo.modarres@gmail.com

Dr_p_salehi@yahoo.com

svallian@sci.ui.ac.ir/sadeqvallian@gmail.com

**ORCID IDs**

Bahareh Maleki: https://orcid.org/0000-0002-3894-7246

Parastoo Modarres: https://orcid.org/0000-0002-7945-4798

Sadeq Vallian: https://orcid.org/0000-0002-5151-5923

**Acknowledgments**

We are grateful to the patients and their family who participated in this study. This research was supported internally by the department of research of the University of Isfahan (Isfahan, Iran).

**Supplementary Table S1:** QRT-PCR-primers data used in this study.

| Gene ID | Definition | NCBI Reference Sequence | Primer sequence (5'→3') | Amplicon (bp) | Ta (ºC) |
| --- | --- | --- | --- | --- | --- |
| *ITPR1* | Inositol 1,4,5-trisphosphate receptor type 1, protein coding | NM_001378452.1 | **F:**TTGCCTCCACAATTCTACGACT  **R:**GGCACAGATCACCAGATACAAC | 200 | 59 |
| *Bcl-2* | B-cell lymphoma 2, anti-apoptotic protein, protein coding | NM_000633.3 | **F:**GGTGAACTGGGGGAGGATTGT  **R:** CTTCAGAGACAGCCAGGAGAA | 232 | 60 |
| *Bax* | Bcl-2-associated X, pro-apoptotic protein, protein coding | NM_[138761.4](https://www.ncbi.nlm.nih.gov/entrez/viewer.fcgi?db=nucleotide&id=1519313979) | **F:** AGAGGATGATTGCCGCCGT  **R:** CAACCACCCTGGTCTTGGATC | 244 | 60 |
| *GAPDH*  (Internal control) | Glyceraldehyde-3-phosphate dehydrogenase, protein coding | [NM_002046.7](https://www.ncbi.nlm.nih.gov/entrez/viewer.fcgi?db=nucleotide&id=1519316078) | **F:** CAGGGCTGCTTTTAACTCTGG  **R:**TGGGTGGAATCATATTGGAACA | 102 | 59 |
| hsa-miR-34b-5p | MicroRNA 34b, ncRNA | NR_029839.1 | **F:**TAGGCAGTGTCATTAGC  **R:** Unknown | **_____** | 60 |
| SNORD-47  (Internal control) | Small nucleolar RNA, C/D box 47, snoRNA | NR_002746.1 | **F:** ATCACTGTAAAACCGTT  **R:** Unknown | **_____** | 60 |

Note: ncRNA, Non-coding RNA; F, Forward primer; R, Reverse primer; Ta, Temperature of annealing

**Supplementary Table S2:** The optimized conditions of qRT-PCR .

| Total volume | Step | Cycle | Time (s) | Temperature (ºC) | | | cDNA (1 µl) |
| --- | --- | --- | --- | --- | --- | --- | --- |
| 15 | Polymerase activation | 1 | 180 | 95 | | | *ITPR1* ^a^  *Bcl-2* ^b^  *Bax* ^c^ |
|  | Denaturation | 1 | 15 | 95 | | |  |
|  | Annealing | 40 | 30 | 60 ^c^ | 60 ^b^ | 59 ^a^ |  |
|  | Extension | 40 | 30 | 72 | | |  |
| 13 | Polymerase activation | 1 | 120 | 95 | | | Hsa-miR-34b-5p |
|  | Denaturation | 40 | 5 | 95 | | |  |
|  | Annealing/ Extension | 40 | 30 | 60 | | |  |

Note: Melting curve calculation following the final protocol: 55ºC to 95ºC in 1ºC increments with samples held at each increment for 5 s.

**Supplementary Table S3:** GEO samples involved in the present study.

| # | Accession | Study | Platform | Group | # | Accession | Study | Platform | Group |
| --- | --- | --- | --- | --- | --- | --- | --- | --- | --- |
| 1 | GSM2915445 | GSE108886 | Illumina | NOA | 51 | GSM233009 | GSE9210 | Agilent | NOA |
| 2 | GSM2915446 | GSE108886 | Illumina | NOA | 52 | GSM233010 | GSE9210 | Agilent | NOA |
| 3 | GSM2915447 | GSE108886 | Illumina | NOA | 53 | GSM233011 | GSE9210 | Agilent | NOA |
| 4 | GSM2915448 | GSE108886 | Illumina | NOA | 54 | GSM233012 | GSE9210 | Agilent | NOA |
| 5 | GSM2915449 | GSE108886 | Illumina | OA | 55 | GSM233013 | GSE9210 | Agilent | NOA |
| 6 | GSM2915450 | GSE108886 | Illumina | NOA | 56 | GSM233014 | GSE9210 | Agilent | NOA |
| 7 | GSM2915451 | GSE108886 | Illumina | OA | 57 | GSM233015 | GSE9210 | Agilent | NOA |
| 8 | GSM2915452 | GSE108886 | Illumina | NOA | 58 | GSM233016 | GSE9210 | Agilent | NOA |
| 9 | GSM2915453 | GSE108886 | Illumina | NOA | 59 | GSM233017 | GSE9210 | Agilent | OA |
| 10 | GSM2915454 | GSE108886 | Illumina | NOA | 60 | GSM233019 | GSE9210 | Agilent | OA |
| 11 | GSM2915455 | GSE108886 | Illumina | OA | 61 | GSM233020 | GSE9210 | Agilent | OA |
| 12 | GSM232970 | GSE9210 | Agilent | NOA | 62 | GSM233021 | GSE9210 | Agilent | OA |
| 13 | GSM232971 | GSE9210 | Agilent | NOA | 63 | GSM233022 | GSE9210 | Agilent | OA |
| 14 | GSM232972 | GSE9210 | Agilent | NOA | 64 | GSM233023 | GSE9210 | Agilent | OA |
| 15 | GSM232973 | GSE9210 | Agilent | NOA | 65 | GSM233024 | GSE9210 | Agilent | OA |
| 16 | GSM232974 | GSE9210 | Agilent | NOA | 66 | GSM233025 | GSE9210 | Agilent | OA |
| 17 | GSM232975 | GSE9210 | Agilent | NOA | 67 | GSM233027 | GSE9210 | Agilent | OA |
| 18 | GSM232976 | GSE9210 | Agilent | NOA | 68 | GSM4318655 | GSE145467 | Agilent | OA |
| 19 | GSM232977 | GSE9210 | Agilent | NOA | 69 | GSM4318656 | GSE145467 | Agilent | OA |
| 20 | GSM232978 | GSE9210 | Agilent | NOA | 70 | GSM4318657 | GSE145467 | Agilent | OA |
| 21 | GSM232979 | GSE9210 | Agilent | NOA | 71 | GSM4318658 | GSE145467 | Agilent | NOA |
| 22 | GSM232980 | GSE9210 | Agilent | NOA | 72 | GSM4318660 | GSE145467 | Agilent | NOA |
| 23 | GSM232981 | GSE9210 | Agilent | NOA | 73 | GSM4318662 | GSE145467 | Agilent | OA |
| 24 | GSM232982 | GSE9210 | Agilent | NOA | 74 | GSM4318665 | GSE145467 | Agilent | OA |
| 25 | GSM232983 | GSE9210 | Agilent | NOA | 75 | GSM4318668 | GSE145467 | Agilent | NOA |
| 26 | GSM232984 | GSE9210 | Agilent | NOA | 76 | GSM4318670 | GSE145467 | Agilent | NOA |
| 27 | GSM232985 | GSE9210 | Agilent | NOA | 77 | GSM4318673 | GSE145467 | Agilent | OA |
| 28 | GSM232986 | GSE9210 | Agilent | NOA | 78 | GSM4318674 | GSE145467 | Agilent | OA |
| 29 | GSM232987 | GSE9210 | Agilent | NOA | 79 | GSM4318675 | GSE145467 | Agilent | NOA |
| 30 | GSM232988 | GSE9210 | Agilent | NOA | 80 | GSM4318676 | GSE145467 | Agilent | OA |
| 31 | GSM232989 | GSE9210 | Agilent | NOA | 81 | GSM4318677 | GSE145467 | Agilent | OA |
| 32 | GSM232990 | GSE9210 | Agilent | NOA | 82 | GSM4318678 | GSE145467 | Agilent | NOA |
| 33 | GSM232991 | GSE9210 | Agilent | NOA | 83 | GSM4318679 | GSE145467 | Agilent | NOA |
| 34 | GSM232992 | GSE9210 | Agilent | NOA | 84 | GSM4318680 | GSE145467 | Agilent | NOA |
| 35 | GSM232993 | GSE9210 | Agilent | NOA | 85 | GSM4318681 | GSE145467 | Agilent | NOA |
| 36 | GSM232994 | GSE9210 | Agilent | NOA | 86 | GSM4318682 | GSE145467 | Agilent | NOA |
| 37 | GSM232995 | GSE9210 | Agilent | NOA | 87 | GSM1118247 | GSE45885 | Affymetrix | NOA |
| 38 | GSM232996 | GSE9210 | Agilent | NOA | 88 | GSM1118248 | GSE45885 | Affymetrix | NOA |
| 39 | GSM232997 | GSE9210 | Agilent | NOA | 89 | GSM1118249 | GSE45885 | Affymetrix | NOA |
| 40 | GSM232998 | GSE9210 | Agilent | NOA | 90 | GSM1118250 | GSE45885 | Affymetrix | NOA |
| 41 | GSM232999 | GSE9210 | Agilent | NOA | 91 | GSM1118258 | GSE45885 | Affymetrix | NOA |
| 42 | GSM233000 | GSE9210 | Agilent | NOA | 92 | GSM1118260 | GSE45885 | Affymetrix | NOA |
| 43 | GSM233001 | GSE9210 | Agilent | NOA | 93 | GSM1118261 | GSE45885 | Affymetrix | NOA |
| 44 | GSM233002 | GSE9210 | Agilent | NOA | 94 | GSM1118262 | GSE45885 | Affymetrix | NOA |
| 45 | GSM233003 | GSE9210 | Agilent | NOA | 95 | GSM1118263 | GSE45885 | Affymetrix | NOA |
| 46 | GSM233004 | GSE9210 | Agilent | NOA | 96 | GSM1118264 | GSE45885 | Affymetrix | NOA |
| 47 | GSM233005 | GSE9210 | Agilent | NOA | 97 | GSM1118266 | GSE45885 | Affymetrix | NOA |
| 48 | GSM233006 | GSE9210 | Agilent | NOA | 98 | GSM1118267 | GSE45885 | Affymetrix | NOA |
| 49 | GSM233007 | GSE9210 | Agilent | NOA | 99 | GSM1118269 | GSE45885 | Affymetrix | NOA |
| 50 | GSM233008 | GSE9210 | Agilent | NOA | 100 | GSM1118270 | GSE45885 | Affymetrix | NOA |
| The sample number of each group: NOA: 79 ; OA:21 | | | | | | | | | |

**Supplementary Table S4:** Differentially expressed genes (DEGs) associated with the non-obstructive azoospermia (NOA).

| # | ID | logFC | P-Value | adj.P-Value | Gene.Symbol |
| --- | --- | --- | --- | --- | --- |
| 1 | 10023 | -2.19372 | 1.48E-15 | 2.85E-14 | FRAT1 |
| 2 | 10062 | -1.3422 | 1.18E-17 | 3.55E-16 | NR1H3 |
| 3 | 1019 | 1.313365 | 9.94E-15 | 1.60E-13 | CDK4 |
| 4 | 10212 | -1.61114 | 4.33E-20 | 2.31E-18 | DDX39 |
| 5 | 1028 | 1.232434 | 4.94E-14 | 6.85E-13 | CDKN1C |
| 6 | 10370 | 1.099083 | 4.84E-17 | 1.28E-15 | CITED2 |
| 7 | 1043 | 1.104928 | 4.26E-13 | 4.71E-12 | CD52 |
| 8 | 10434 | -1.27006 | 8.28E-24 | 1.46E-21 | LYPLA1 |
| 9 | 10536 | 2.026506 | 2.07E-20 | 1.20E-18 | LEPREL2 |
| 10 | 10610 | -1.61565 | 1.36E-26 | 4.04E-24 | ST6GALNAC2 |
| 11 | 10615 | -2.36603 | 3.78E-22 | 4.27E-20 | SPAG5 |
| 12 | 10656 | -2.12351 | 2.42E-12 | 2.31E-11 | KHDRBS3 |
| 13 | 10665 | -2.76259 | 1.68E-21 | 1.35E-19 | C6orf10 |
| 14 | 10732 | -1.62242 | 2.23E-20 | 1.27E-18 | TCFL5 |
| 15 | 10744 | -1.4647 | 4.74E-18 | 1.58E-16 | PTTG2 |
| 16 | 10807 | -1.40623 | 9.45E-27 | 3.13E-24 | SDCCAG3 |
| 17 | 10809 | -1.376 | 8.17E-14 | 1.10E-12 | STARD10 |
| 18 | 10880 | -2.95311 | 3.20E-12 | 2.97E-11 | ACTL7B |
| 19 | 10881 | -3.74679 | 3.12E-15 | 5.59E-14 | ACTL7A |
| 20 | 11092 | -2.89047 | 1.36E-17 | 4.06E-16 | C9orf9 |
| 21 | 11104 | -1.49881 | 2.32E-19 | 1.09E-17 | KATNA1 |
| 22 | 11124 | -1.19876 | 8.46E-28 | 4.33E-25 | FAF1 |
| 23 | 11127 | -1.11131 | 7.47E-16 | 1.53E-14 | KIF3A |
| 24 | 11218 | -2.35831 | 6.55E-20 | 3.26E-18 | DDX20 |
| 25 | 112401 | -1.78639 | 8.42E-15 | 1.38E-13 | BIRC8 |
| 26 | 113263 | -1.27769 | 8.45E-18 | 2.63E-16 | GLCCI1 |
| 27 | 114327 | -1.53827 | 1.74E-24 | 3.39E-22 | EFHC1 |
| 28 | 114659 | -1.76734 | 6.12E-22 | 5.94E-20 | LRRC37B |
| 29 | 115509 | -1.38151 | 2.28E-13 | 2.73E-12 | ZNF689 |
| 30 | 115811 | -2.83068 | 1.57E-19 | 7.56E-18 | IQCD |
| 31 | 1164 | -1.65445 | 1.25E-12 | 1.27E-11 | CKS2 |
| 32 | 116832 | -2.20475 | 3.17E-15 | 5.66E-14 | RPL39L |
| 33 | 116969 | -2.50051 | 5.54E-13 | 6.03E-12 | ART5 |
| 34 | 117155 | -1.85615 | 2.45E-14 | 3.66E-13 | CATSPER2 |
| 35 | 117177 | -1.27606 | 3.91E-21 | 2.78E-19 | RAB3IP |
| 36 | 118429 | 1.260595 | 1.96E-13 | 2.38E-12 | ANTXR2 |
| 37 | 119180 | -3.95775 | 3.19E-17 | 8.67E-16 | LYZL2 |
| 38 | 121355 | -2.59921 | 6.28E-15 | 1.06E-13 | FAM112B |
| 39 | 122664 | -3.54224 | 1.36E-18 | 5.27E-17 | C14orf8 |
| 40 | 124626 | -3.46203 | 6.13E-12 | 5.42E-11 | ZPBP2 |
| 41 | 124783 | -2.68364 | 3.62E-16 | 7.83E-15 | C17orf46 |
| 42 | 124912 | -3.52827 | 6.20E-19 | 2.64E-17 | SPACA3 |
| 43 | 125875 | -1.84037 | 1.66E-15 | 3.15E-14 | CLDND2 |
| 44 | 126823 | -3.10814 | 1.90E-13 | 2.32E-12 | KARCA1 |
| 45 | 128602 | -3.52278 | 9.97E-13 | 1.02E-11 | C20orf85 |
| 46 | 128653 | -1.42511 | 1.15E-11 | 9.51E-11 | C20orf141 |
| 47 | 130540 | -1.83395 | 2.98E-22 | 3.57E-20 | ALS2CR12 |
| 48 | 130814 | 1.13296 | 2.84E-14 | 4.20E-13 | PQLC3 |
| 49 | 131544 | -1.03667 | 1.81E-16 | 4.08E-15 | DKFZp667G2110 |
| 50 | 131965 | -1.11345 | 4.78E-27 | 1.92E-24 | METTL6 |
| 51 | 132141 | -2.86654 | 1.29E-20 | 7.89E-19 | IQCF1 |
| 52 | 132612 | -4.82982 | 1.60E-14 | 2.49E-13 | Tenr |
| 53 | 132671 | -1.77053 | 3.03E-13 | 3.49E-12 | SPATA18 |
| 54 | 135927 | -2.2865 | 3.25E-18 | 1.14E-16 | C7orf34 |
| 55 | 136541 | -3.03743 | 5.66E-19 | 2.43E-17 | TRY1 |
| 56 | 137392 | -2.48481 | 1.93E-27 | 9.06E-25 | FAM92A1 |
| 57 | 1375 | -2.22483 | 5.70E-20 | 2.95E-18 | CPT1B |
| 58 | 137994 | -1.39253 | 6.39E-15 | 1.08E-13 | LETM2 |
| 59 | 138162 | -1.7175 | 2.92E-15 | 5.30E-14 | C9orf116 |
| 60 | 140290 | -3.72353 | 5.72E-18 | 1.86E-16 | TCP10L |
| 61 | 140735 | -2.46716 | 3.09E-20 | 1.69E-18 | DYNLL2 |
| 62 | 140836 | -2.77613 | 4.77E-15 | 8.18E-14 | C20orf179 |
| 63 | 1434 | -1.05442 | 6.49E-13 | 6.94E-12 | CSE1L |
| 64 | 144406 | -2.00359 | 3.11E-14 | 4.52E-13 | WDR66 |
| 65 | 1459 | -1.20377 | 3.09E-14 | 4.49E-13 | CSNK2A2 |
| 66 | 145942 | -4.19329 | 2.66E-21 | 2.02E-19 | TMCO5 |
| 67 | 147184 | -1.60098 | 6.01E-13 | 6.51E-12 | TMEM99 |
| 68 | 147323 | -1.57179 | 1.10E-11 | 9.19E-11 | STARD6 |
| 69 | 1490 | 1.296806 | 6.40E-13 | 6.86E-12 | CTGF |
| 70 | 149018 | -3.63039 | 3.04E-18 | 1.09E-16 | LELP1 |
| 71 | 150291 | -2.8107 | 5.70E-13 | 6.19E-12 | C22orf27 |
| 72 | 151649 | -2.63636 | 3.79E-22 | 4.27E-20 | C3orf48 |
| 73 | 151827 | -2.33525 | 6.74E-27 | 2.37E-24 | LRRC34 |
| 74 | 152007 | 1.459085 | 2.14E-24 | 4.02E-22 | C9orf19 |
| 75 | 152405 | -2.93872 | 2.90E-17 | 8.02E-16 | C3orf30 |
| 76 | 153733 | -1.72079 | 4.32E-21 | 3.04E-19 | CCDC112 |
| 77 | 157869 | -1.44422 | 1.57E-17 | 4.58E-16 | RPESP |
| 78 | 162333 | -1.65547 | 7.34E-13 | 7.74E-12 | RNF190 |
| 79 | 162517 | -2.54726 | 9.16E-19 | 3.68E-17 | FBXO39 |
| 80 | 163 | -1.11237 | 4.39E-12 | 3.95E-11 | AP2B1 |
| 81 | 163059 | -1.74592 | 1.39E-17 | 4.12E-16 | ZNF433 |
| 82 | 1635 | 1.113615 | 2.00E-16 | 4.46E-15 | DCTD |
| 83 | 165721 | -3.3804 | 5.46E-18 | 1.79E-16 | DNAJB8 |
| 84 | 1769 | -1.93482 | 1.57E-19 | 7.56E-18 | DNAH8 |
| 85 | 1947 | 1.212088 | 1.16E-17 | 3.51E-16 | EFNB1 |
| 86 | 196743 | -2.18113 | 6.75E-13 | 7.15E-12 | PAOX |
| 87 | 199223 | -3.29582 | 1.06E-13 | 1.37E-12 | TTC21A |
| 88 | 2013 | 1.457922 | 7.42E-13 | 7.79E-12 | EMP2 |
| 89 | 219743 | -1.65399 | 3.32E-17 | 8.98E-16 | TYSND1 |
| 90 | 219771 | -1.59751 | 1.08E-15 | 2.14E-14 | C10orf9 |
| 91 | 2200 | 1.344893 | 4.27E-14 | 6.00E-13 | FBN1 |
| 92 | 220972 | -2.23576 | 3.48E-15 | 6.16E-14 | 8-Mar |
| 93 | 221908 | -1.46798 | 7.53E-17 | 1.89E-15 | MGC22793 |
| 94 | 222698 | -2.21061 | 3.34E-21 | 2.44E-19 | C6orf194 |
| 95 | 22873 | -2.04443 | 2.87E-23 | 4.15E-21 | DZIP1 |
| 96 | 2290 | -1.46683 | 1.41E-12 | 1.43E-11 | FOXG1B |
| 97 | 22921 | 1.084961 | 1.49E-21 | 1.24E-19 | MSRB2 |
| 98 | 23059 | -1.08636 | 3.92E-13 | 4.41E-12 | CLUAP1 |
| 99 | 23113 | -1.33684 | 1.41E-17 | 4.14E-16 | PARC |
| 100 | 23136 | -1.99539 | 1.56E-12 | 1.56E-11 | EPB41L3 |
| 101 | 23324 | 1.019637 | 3.73E-15 | 6.53E-14 | MAN2B2 |
| 102 | 23397 | -1.47129 | 8.10E-16 | 1.65E-14 | BRRN1 |
| 103 | 23424 | -1.7695 | 5.01E-16 | 1.06E-14 | TDRD7 |
| 104 | 23617 | -3.39323 | 1.47E-16 | 3.42E-15 | TSSK2 |
| 105 | 2519 | 1.58081 | 3.34E-15 | 5.93E-14 | FUCA2 |
| 106 | 255762 | -1.97042 | 6.86E-25 | 1.49E-22 | C16orf65 |
| 107 | 256957 | -1.79354 | 7.35E-15 | 1.22E-13 | C17orf66 |
| 108 | 257236 | -2.76049 | 7.13E-14 | 9.70E-13 | CCDC96 |
| 109 | 25886 | -1.00449 | 2.16E-14 | 3.26E-13 | WDR51A |
| 110 | 25888 | -1.75191 | 1.26E-13 | 1.60E-12 | ZNF473 |
| 111 | 25896 | -1.30217 | 2.91E-21 | 2.18E-19 | INTS7 |
| 112 | 25904 | -1.02465 | 4.70E-24 | 8.53E-22 | CNOT10 |
| 113 | 25911 | -2.07908 | 5.15E-22 | 5.18E-20 | RP11-529I10.4 |
| 114 | 25915 | -1.95215 | 5.66E-14 | 7.79E-13 | C3orf60 |
| 115 | 25959 | 1.214759 | 1.12E-16 | 2.66E-15 | ANKRD25 |
| 116 | 26005 | -1.02738 | 5.80E-18 | 1.88E-16 | DKFZP586P0123 |
| 117 | 26040 | 1.161631 | 1.18E-13 | 1.52E-12 | SETBP1 |
| 118 | 26206 | -2.29121 | 2.21E-13 | 2.65E-12 | SPAG8 |
| 119 | 26261 | -3.13083 | 1.24E-14 | 1.98E-13 | FBXO24 |
| 120 | 26292 | -1.40865 | 2.87E-19 | 1.31E-17 | MYCBP |
| 121 | 2635 | 1.389045 | 3.49E-12 | 3.19E-11 | GBP3 |
| 122 | 26499 | 1.349798 | 3.22E-12 | 2.99E-11 | PLEK2 |
| 123 | 26608 | -2.29658 | 2.19E-12 | 2.10E-11 | TBL2 |
| 124 | 266747 | -1.45674 | 1.82E-21 | 1.45E-19 | RGR |
| 125 | 27074 | -2.06817 | 1.55E-19 | 7.56E-18 | LAMP3 |
| 126 | 2720 | 1.12619 | 1.68E-17 | 4.87E-16 | GLB1 |
| 127 | 283 | 1.006392 | 1.70E-12 | 1.68E-11 | ANG |
| 128 | 283254 | -1.17634 | 2.21E-13 | 2.65E-12 | C11orf77 |
| 129 | 283385 | -2.28185 | 1.25E-15 | 2.45E-14 | MORN3 |
| 130 | 284382 | -2.54006 | 2.71E-13 | 3.15E-12 | MGC33407 |
| 131 | 284403 | -1.38503 | 2.35E-13 | 2.80E-12 | WDR62 |
| 132 | 285148 | -1.21512 | 3.07E-15 | 5.52E-14 | LOC285148 |
| 133 | 285362 | 1.013923 | 2.89E-17 | 8.02E-16 | SUMF1 |
| 134 | 2869 | 1.434447 | 3.40E-12 | 3.13E-11 | GRK5 |
| 135 | 29098 | -1.53154 | 3.74E-19 | 1.66E-17 | RANGNRF |
| 136 | 29098 | -1.53154 | 3.74E-19 | 1.66E-17 | RANGNRF |
| 137 | 29103 | -1.31078 | 1.75E-12 | 1.73E-11 | DNAJC15 |
| 138 | 29119 | -1.05246 | 8.63E-17 | 2.12E-15 | CTNNA3 |
| 139 | 29799 | -2.54182 | 5.98E-17 | 1.54E-15 | YPEL1 |
| 140 | 29843 | -1.74908 | 2.44E-17 | 6.88E-16 | SENP1 |
| 141 | 29978 | 1.41385 | 4.15E-19 | 1.82E-17 | UBQLN2 |
| 142 | 30815 | 1.187706 | 5.36E-13 | 5.85E-12 | ST6GALNAC6 |
| 143 | 3092 | -1.32851 | 1.87E-12 | 1.83E-11 | HIP1 |
| 144 | 3098 | -1.33664 | 1.60E-21 | 1.31E-19 | HK1 |
| 145 | 3109 | 1.305672 | 1.84E-13 | 2.26E-12 | HLA-DMB |
| 146 | 317719 | -2.93409 | 1.17E-15 | 2.29E-14 | KLHL10 |
| 147 | 3240 | 1.778915 | 7.57E-14 | 1.02E-12 | HP |
| 148 | 3305 | -2.86873 | 1.31E-16 | 3.09E-15 | HSPA1L |
| 149 | 3338 | -1.18734 | 2.31E-13 | 2.75E-12 | DNAJC4 |
| 150 | 3416 | -1.37236 | 6.73E-27 | 2.37E-24 | IDE |
| 151 | 3487 | 1.754002 | 1.33E-12 | 1.35E-11 | IGFBP4 |
| 152 | 348793 | -2.14558 | 1.21E-23 | 2.07E-21 | WDR53 |
| 153 | 3488 | 1.527407 | 7.17E-13 | 7.57E-12 | IGFBP5 |
| 154 | 3628 | -1.95016 | 2.36E-21 | 1.84E-19 | INPP1 |
| 155 | 3706 | -1.06523 | 8.39E-19 | 3.42E-17 | ITPKA |
| 156 | 3708 | 1.336552 | 3.37E-12 | 3.12E-11 | ITPR1 |
| 157 | 3796 | -1.30826 | 2.03E-20 | 1.19E-18 | KIF2 |
| 158 | 3838 | -1.90233 | 5.47E-21 | 3.76E-19 | KPNA2 |
| 159 | 3927 | 1.322827 | 9.51E-14 | 1.24E-12 | LASP1 |
| 160 | 3959 | 1.005799 | 4.54E-18 | 1.52E-16 | LGALS3BP |
| 161 | 3988 | 1.14076 | 4.47E-12 | 4.01E-11 | LIPA |
| 162 | 4055 | 1.125119 | 3.13E-18 | 1.12E-16 | LTBR |
| 163 | 4060 | 2.295444 | 1.62E-12 | 1.62E-11 | LUM |
| 164 | 4067 | 1.416899 | 3.03E-14 | 4.43E-13 | LYN |
| 165 | 4088 | 1.217018 | 1.53E-13 | 1.91E-12 | SMAD3 |
| 166 | 4094 | 1.2104 | 4.67E-12 | 4.18E-11 | MAF |
| 167 | 4478 | 1.605598 | 4.04E-18 | 1.38E-16 | MSN |
| 168 | 4603 | -1.021 | 6.85E-12 | 5.96E-11 | MYBL1 |
| 169 | 4609 | 1.011888 | 4.39E-15 | 7.58E-14 | MYC |
| 170 | 4774 | 1.209224 | 3.40E-12 | 3.13E-11 | NFIA |
| 171 | 4781 | 1.024892 | 1.57E-13 | 1.95E-12 | NFIB |
| 172 | 4841 | 1.152295 | 2.53E-18 | 9.32E-17 | NONO |
| 173 | 4898 | -1.05685 | 6.24E-29 | 7.02E-26 | NRD1 |
| 174 | 4927 | -1.6288 | 1.10E-19 | 5.46E-18 | NUP88 |
| 175 | 4956 | -3.7605 | 4.27E-14 | 6.00E-13 | ODF1 |
| 176 | 50619 | -2.08033 | 3.78E-44 | 2.13E-40 | DEF6 |
| 177 | 50628 | -1.52315 | 1.86E-16 | 4.18E-15 | GEMIN4 |
| 178 | 50855 | -1.86581 | 1.86E-18 | 7.07E-17 | PARD6A |
| 179 | 51009 | -1.03194 | 3.91E-16 | 8.37E-15 | DERL2 |
| 180 | 51019 | -1.49787 | 3.15E-15 | 5.63E-14 | CCDC53 |
| 181 | 51066 | -1.38349 | 1.48E-15 | 2.85E-14 | C3orf32 |
| 182 | 51072 | -1.22714 | 6.15E-23 | 8.45E-21 | C2orf4 |
| 183 | 51097 | -2.14718 | 1.29E-13 | 1.63E-12 | SCCPDH |
| 184 | 5110 | -1.0256 | 3.47E-18 | 1.21E-16 | PCMT1 |
| 185 | 51100 | -1.84283 | 1.72E-17 | 4.97E-16 | SH3GLB1 |
| 186 | 51127 | -2.30282 | 1.53E-18 | 5.92E-17 | TRIM17 |
| 187 | 51136 | -2.12072 | 2.17E-13 | 2.62E-12 | LOC51136 |
| 188 | 51167 | -1.56385 | 7.24E-26 | 1.79E-23 | CYB5R4 |
| 189 | 51265 | -1.02328 | 6.60E-19 | 2.79E-17 | CDKL3 |
| 190 | 51313 | 1.320936 | 1.63E-13 | 2.02E-12 | C4orf18 |
| 191 | 51444 | -1.71663 | 1.47E-13 | 1.84E-12 | RNF138 |
| 192 | 51460 | -1.75674 | 1.76E-15 | 3.32E-14 | SFMBT1 |
| 193 | 51593 | -1.03546 | 9.39E-17 | 2.29E-15 | ARS2 |
| 194 | 51692 | -1.13372 | 5.02E-22 | 5.18E-20 | CPSF3 |
| 195 | 53340 | -3.94792 | 4.32E-20 | 2.31E-18 | SPA17 |
| 196 | 5351 | 1.578363 | 2.68E-12 | 2.54E-11 | PLOD1 |
| 197 | 54148 | -1.01359 | 1.36E-26 | 4.04E-24 | MRPL39 |
| 198 | 5423 | -1.41423 | 2.42E-26 | 6.80E-24 | POLB |
| 199 | 54503 | -1.02882 | 6.17E-20 | 3.13E-18 | ZDHHC13 |
| 200 | 54535 | -1.32165 | 2.85E-19 | 1.31E-17 | CCHCR1 |
| 201 | 54558 | -4.23282 | 4.36E-18 | 1.47E-16 | SPATA6 |
| 202 | 54585 | -1.81757 | 2.56E-19 | 1.19E-17 | LZTFL1 |
| 203 | 5480 | 1.172027 | 6.29E-16 | 1.30E-14 | PPIC |
| 204 | 54879 | -1.3538 | 2.67E-15 | 4.90E-14 | ST7L |
| 205 | 54913 | 1.333923 | 2.12E-12 | 2.05E-11 | RPP25 |
| 206 | 54920 | -1.10256 | 1.34E-17 | 4.00E-16 | DUS2L |
| 207 | 54937 | -1.6347 | 2.46E-13 | 2.89E-12 | SOHLH2 |
| 208 | 54997 | -1.2531 | 9.97E-16 | 1.99E-14 | TESC |
| 209 | 54998 | -1.46393 | 2.91E-18 | 1.05E-16 | AURKAIP1 |
| 210 | 55012 | -1.54287 | 3.04E-29 | 4.28E-26 | C14orf10 |
| 211 | 55064 | -2.25398 | 2.86E-13 | 3.31E-12 | C9orf68 |
| 212 | 5510 | -1.7293 | 2.47E-18 | 9.16E-17 | PPP1R7 |
| 213 | 55112 | -1.23145 | 3.19E-26 | 8.55E-24 | WDR60 |
| 214 | 55132 | -1.48656 | 1.69E-23 | 2.57E-21 | LARP2 |
| 215 | 55148 | -1.8918 | 2.67E-28 | 1.88E-25 | C14orf130 |
| 216 | 55256 | 1.097439 | 2.94E-14 | 4.33E-13 | ADI1 |
| 217 | 55323 | 1.053097 | 2.97E-13 | 3.43E-12 | LARP6 |
| 218 | 55333 | -1.05074 | 4.91E-13 | 5.41E-12 | SYNJ2BP |
| 219 | 55342 | -1.2324 | 4.08E-15 | 7.09E-14 | STRBP |
| 220 | 55352 | -2.10642 | 6.92E-18 | 2.19E-16 | C17orf79 |
| 221 | 55471 | -2.21097 | 1.14E-21 | 1.00E-19 | PRO1853 |
| 222 | 55603 | 1.405225 | 3.98E-14 | 5.64E-13 | FAM46A |
| 223 | 5562 | -1.87782 | 7.28E-17 | 1.86E-15 | PRKAA1 |
| 224 | 55632 | -1.39874 | 1.89E-22 | 2.32E-20 | KIAA1333 |
| 225 | 55732 | -1.08196 | 1.56E-13 | 1.94E-12 | C1orf112 |
| 226 | 55805 | -1.43571 | 5.92E-18 | 1.91E-16 | LRP2BP |
| 227 | 55812 | -2.05914 | 1.49E-21 | 1.24E-19 | SPATA7 |
| 228 | 55818 | -1.09975 | 6.08E-23 | 8.45E-21 | JMJD1A |
| 229 | 55827 | -1.68697 | 2.23E-20 | 1.27E-18 | IQWD1 |
| 230 | 5596 | 1.205348 | 9.94E-16 | 1.99E-14 | MAPK4 |
| 231 | 56269 | -2.28638 | 1.35E-13 | 1.71E-12 | IRGC |
| 232 | 5654 | 1.676499 | 4.24E-15 | 7.34E-14 | HTRA1 |
| 233 | 5687 | -1.55017 | 1.61E-35 | 4.53E-32 | PSMA6 |
| 234 | 56942 | -1.1358 | 9.94E-19 | 3.94E-17 | C16orf61 |
| 235 | 56967 | 1.296064 | 3.40E-12 | 3.13E-11 | C14orf132 |
| 236 | 57149 | -1.31148 | 1.20E-20 | 7.48E-19 | LOC57149 |
| 237 | 57151 | -2.80215 | 5.75E-17 | 1.50E-15 | LYZL6 |
| 238 | 57231 | -1.25553 | 1.26E-20 | 7.81E-19 | SNX14 |
| 239 | 57639 | -1.84841 | 7.24E-16 | 1.49E-14 | KIAA1505 |
| 240 | 5833 | -1.61129 | 7.42E-15 | 1.23E-13 | PCYT2 |
| 241 | 59274 | 1.128274 | 3.76E-14 | 5.36E-13 | MESDC1 |
| 242 | 5990 | -2.70374 | 4.31E-14 | 6.03E-13 | RFX2 |
| 243 | 5992 | -1.52065 | 5.89E-12 | 5.23E-11 | RFX4 |
| 244 | 60386 | -1.37705 | 6.43E-15 | 1.08E-13 | SLC25A19 |
| 245 | 64232 | -3.21408 | 5.79E-17 | 1.50E-15 | MS4A5 |
| 246 | 64333 | -1.65021 | 2.54E-14 | 3.78E-13 | ARHGAP9 |
| 247 | 6442 | -1.70235 | 1.82E-13 | 2.24E-12 | SGCA |
| 248 | 6448 | 1.22101 | 1.20E-21 | 1.04E-19 | SGSH |
| 249 | 6457 | -2.90276 | 2.43E-21 | 1.87E-19 | SH3GL3 |
| 250 | 6464 | 1.141825 | 1.04E-18 | 4.09E-17 | SHC1 |
| 251 | 65082 | -1.00628 | 1.09E-18 | 4.25E-17 | VPS33A |
| 252 | 6567 | 1.406757 | 2.47E-13 | 2.90E-12 | SLC16A2 |
| 253 | 65990 | -1.03714 | 6.79E-14 | 9.25E-13 | C16orf24 |
| 254 | 6610 | -1.83383 | 1.29E-16 | 3.05E-15 | SMPD2 |
| 255 | 6775 | -2.05366 | 4.11E-22 | 4.45E-20 | STAT4 |
| 256 | 6835 | -1.58299 | 8.75E-19 | 3.55E-17 | SURF2 |
| 257 | 6881 | -1.11047 | 7.94E-15 | 1.30E-13 | TAF10 |
| 258 | 6909 | 1.805671 | 5.98E-22 | 5.91E-20 | TBX2 |
| 259 | 6923 | -1.099 | 1.25E-13 | 1.60E-12 | TCEB2 |
| 260 | 7045 | 1.867362 | 6.57E-17 | 1.68E-15 | TGFBI |
| 261 | 706 | 1.445802 | 4.26E-19 | 1.86E-17 | TSPO |
| 262 | 7142 | -2.84582 | 1.64E-14 | 2.53E-13 | TNP2 |
| 263 | 7169 | 1.186302 | 2.36E-15 | 4.36E-14 | TPM2 |
| 264 | 7180 | -4.697 | 1.78E-18 | 6.81E-17 | CRISP2 |
| 265 | 7216 | 1.048104 | 1.07E-11 | 8.97E-11 | TRO |
| 266 | 7247 | -1.28583 | 4.00E-18 | 1.37E-16 | TSN |
| 267 | 7277 | -1.19716 | 1.61E-12 | 1.60E-11 | TUBA1 |
| 268 | 7277 | -1.19716 | 1.61E-12 | 1.60E-11 | TUBA1 |
| 269 | 7288 | -2.34224 | 4.53E-12 | 4.07E-11 | TULP2 |
| 270 | 7402 | -1.53421 | 8.58E-13 | 8.90E-12 | UTRN |
| 271 | 7423 | 1.496262 | 2.23E-14 | 3.35E-13 | VEGFB |
| 272 | 79085 | 1.156418 | 4.70E-12 | 4.20E-11 | SLC25A23 |
| 273 | 79365 | 1.053614 | 4.72E-14 | 6.58E-13 | BHLHB3 |
| 274 | 79443 | 1.247763 | 1.14E-21 | 1.00E-19 | FYCO1 |
| 275 | 79632 | -1.14137 | 1.14E-22 | 1.49E-20 | C6orf60 |
| 276 | 79639 | -1.11737 | 9.72E-12 | 8.25E-11 | TMEM53 |
| 277 | 79651 | 1.522462 | 3.31E-14 | 4.77E-13 | RHBDF2 |
| 278 | 79741 | -2.41056 | 1.93E-23 | 2.86E-21 | C10orf68 |
| 279 | 79748 | -2.0408 | 1.74E-12 | 1.72E-11 | LMAN1L |
| 280 | 79789 | -1.43432 | 7.65E-22 | 7.30E-20 | CLMN |
| 281 | 79802 | -1.40617 | 5.79E-15 | 9.84E-14 | KIAA1822L |
| 282 | 79833 | -1.09413 | 2.14E-27 | 9.27E-25 | GEMIN6 |
| 283 | 79865 | -1.45591 | 5.09E-22 | 5.18E-20 | TREML2 |
| 284 | 80173 | -1.28828 | 1.55E-22 | 1.94E-20 | IFT74 |
| 285 | 80199 | -1.19526 | 6.46E-12 | 5.67E-11 | FUZ |
| 286 | 80237 | -1.96703 | 1.64E-15 | 3.11E-14 | ELL3 |
| 287 | 80318 | -3.57826 | 3.57E-19 | 1.61E-17 | GKAP1 |
| 288 | 80821 | -1.26303 | 7.39E-17 | 1.87E-15 | DDHD1 |
| 289 | 81544 | -1.12058 | 9.87E-15 | 1.59E-13 | GDPD5 |
| 290 | 81570 | -2.3101 | 1.11E-16 | 2.66E-15 | CLPB |
| 291 | 81873 | -1.03228 | 3.30E-13 | 3.77E-12 | ARPC5L |
| 292 | 81888 | 1.332529 | 1.12E-13 | 1.44E-12 | HYI |
| 293 | 8220 | -1.17062 | 2.33E-12 | 2.23E-11 | DGCR14 |
| 294 | 8263 | 1.271617 | 2.69E-16 | 5.95E-15 | F8A1 |
| 295 | 832 | -1.23869 | 3.69E-12 | 3.36E-11 | CAPZB |
| 296 | 8331 | -1.28253 | 7.69E-28 | 4.33E-25 | HIST1H2AJ |
| 297 | 8332 | -1.08059 | 6.07E-15 | 1.03E-13 | HIST1H2AL |
| 298 | 83464 | -2.87885 | 1.05E-11 | 8.84E-11 | APH1B |
| 299 | 83538 | -3.63416 | 5.16E-19 | 2.24E-17 | TTC25 |
| 300 | 83657 | -5.11353 | 7.92E-14 | 1.06E-12 | DYNLRB2 |
| 301 | 8372 | -1.1063 | 9.66E-21 | 6.18E-19 | HYAL3 |
| 302 | 83737 | -1.25428 | 3.25E-19 | 1.48E-17 | ITCH |
| 303 | 83853 | -4.70691 | 8.19E-14 | 1.10E-12 | ROPN1L |
| 304 | 83861 | -2.23907 | 1.02E-21 | 9.25E-20 | RSHL2 |
| 305 | 83942 | -3.51675 | 3.36E-18 | 1.17E-16 | TSSK1 |
| 306 | 84218 | -1.21669 | 3.63E-17 | 9.79E-16 | TBC1D3 |
| 307 | 84328 | -1.2024 | 1.04E-16 | 2.51E-15 | LZIC |
| 308 | 84517 | -3.00728 | 2.91E-12 | 2.73E-11 | ARPM1 |
| 309 | 84519 | -3.97576 | 5.35E-13 | 5.85E-12 | ACRBP |
| 310 | 84677 | -2.5124 | 4.08E-15 | 7.09E-14 | DSCR8 |
| 311 | 847 | 1.18163 | 8.88E-13 | 9.20E-12 | CAT |
| 312 | 84890 | -1.22724 | 1.94E-18 | 7.35E-17 | C10orf22 |
| 313 | 84923 | -1.95443 | 1.36E-24 | 2.74E-22 | FAM104A |
| 314 | 8519 | 1.30737 | 1.21E-14 | 1.93E-13 | IFITM1 |
| 315 | 85376 | -3.40867 | 6.24E-18 | 2.00E-16 | KIAA1666 |
| 316 | 85438 | -2.87405 | 1.81E-17 | 5.19E-16 | NYD-SP26 |
| 317 | 8632 | -1.90174 | 9.43E-16 | 1.90E-14 | DNAH17 |
| 318 | 873 | 1.22672 | 4.17E-14 | 5.89E-13 | CBR1 |
| 319 | 874 | 1.252461 | 1.42E-12 | 1.43E-11 | CBR3 |
| 320 | 8798 | -1.18966 | 1.08E-17 | 3.28E-16 | DYRK4 |
| 321 | 881 | -3.35532 | 2.70E-18 | 9.89E-17 | CCIN |
| 322 | 8825 | -1.65634 | 7.56E-18 | 2.38E-16 | LIN7A |
| 323 | 8852 | -3.79997 | 2.00E-17 | 5.68E-16 | AKAP4 |
| 324 | 8853 | -1.60607 | 4.35E-20 | 2.31E-18 | DDEF2 |
| 325 | 8882 | -1.06601 | 6.44E-20 | 3.24E-18 | ZNF259 |
| 326 | 89849 | 1.364411 | 8.68E-12 | 7.42E-11 | ATG16L2 |
| 327 | 89894 | -1.53778 | 8.39E-14 | 1.12E-12 | TMEM116 |
| 328 | 902 | -1.32204 | 4.68E-25 | 1.05E-22 | CCNH |
| 329 | 90423 | -2.53003 | 9.68E-22 | 9.08E-20 | ATP6V1E2 |
| 330 | 90506 | -2.64697 | 2.27E-13 | 2.72E-12 | LRRC46 |
| 331 | 90957 | -1.02477 | 8.52E-17 | 2.10E-15 | DHX57 |
| 332 | 9098 | -1.10274 | 8.42E-14 | 1.12E-12 | USP6 |
| 333 | 9130 | 1.092668 | 2.93E-13 | 3.38E-12 | FAM50A |
| 334 | 9133 | -1.81151 | 7.52E-23 | 1.01E-20 | CCNB2 |
| 335 | 9232 | -2.28515 | 7.85E-25 | 1.64E-22 | PTTG1 |
| 336 | 92749 | -3.50233 | 2.48E-15 | 4.57E-14 | C2orf39 |
| 337 | 92906 | -1.23704 | 1.74E-28 | 1.40E-25 | HNRPLL |
| 338 | 9296 | -1.06865 | 4.68E-20 | 2.46E-18 | ATP6V1F |
| 339 | 9319 | -1.18004 | 3.65E-12 | 3.33E-11 | TRIP13 |
| 340 | 93974 | -1.63314 | 1.52E-23 | 2.44E-21 | ATPIF1 |
| 341 | 94086 | -2.68901 | 1.12E-15 | 2.21E-14 | HSPB9 |
| 342 | 9516 | 1.586694 | 5.77E-14 | 7.91E-13 | LITAF |
| 343 | 9519 | -2.49941 | 1.82E-20 | 1.09E-18 | TBPL1 |
| 344 | 95681 | -1.50792 | 4.39E-16 | 9.33E-15 | TSGA14 |
| 345 | 9631 | -2.00386 | 1.11E-28 | 1.04E-25 | NUP155 |
| 346 | 9636 | 1.143307 | 1.42E-14 | 2.24E-13 | ISG15 |
| 347 | 9644 | 1.563525 | 1.48E-22 | 1.90E-20 | SH3PXD2A |
| 348 | 9650 | -1.15726 | 1.59E-16 | 3.62E-15 | MTFR1 |
| 349 | 9687 | 1.412462 | 1.72E-14 | 2.66E-13 | GREB1 |
| 350 | 995 | -1.39529 | 4.01E-14 | 5.67E-13 | CDC25C |
| 351 | 810 | -1.1307581 | 3.25E-10 | 2.03E-09 | CALML3 |
| 352 | 5568 | -2.91793 | 8.51E-11 | 5.89E-10 | PRKACG |


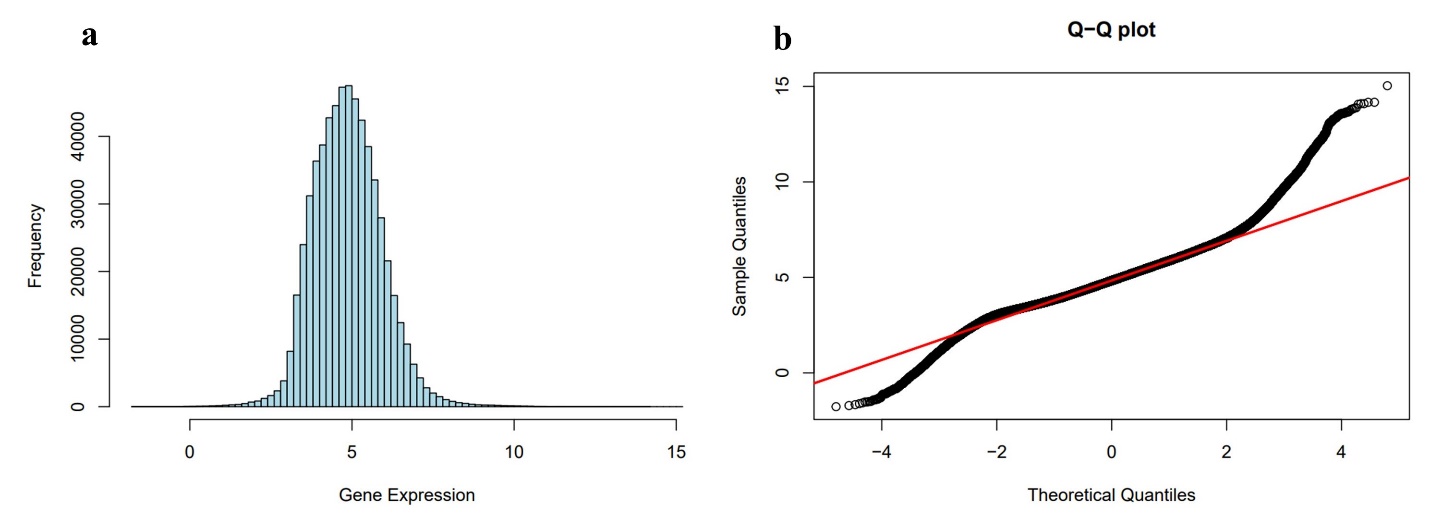
**Supplementary Fig. S1:** Evaluation of normality distribution of statistical data using Histogram and quantile–quantile (Q-Q) Plot. Both a) histogram and b) Q-Q plot of the gene expression profiles show the normal distribution of statistical data.


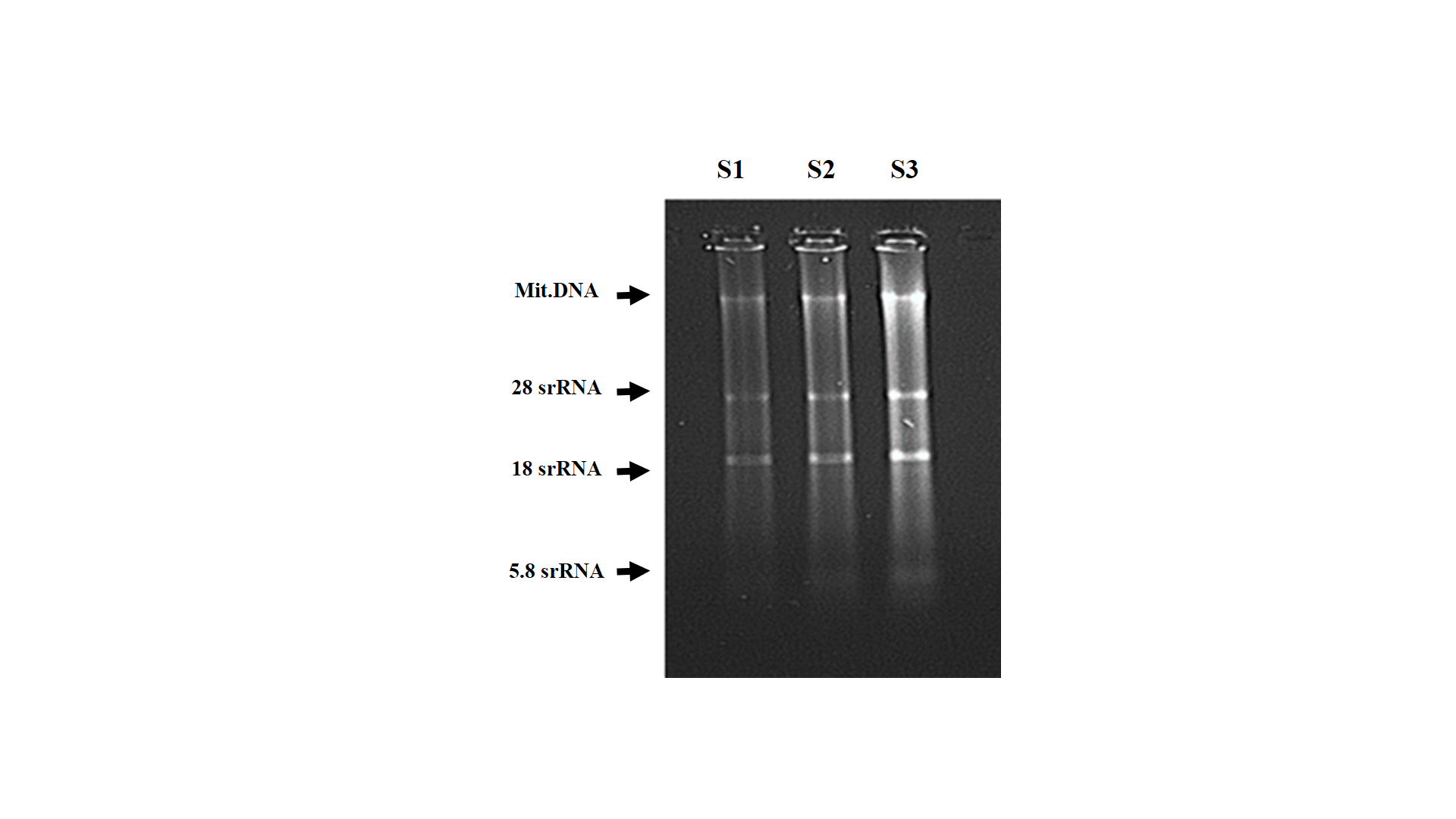
**Supplementary Fig. S2:** **Evaluation of RNA integrity in testis samples by 1.5% denaturing agarose gel electrophoresis.** Bands of 28srRNA, 18srRNA, and 5.8srRNA were placed in order from top to bottom, confirming the high integrity of the extracted RNA as well as the absence of additional bands in this study. S Samples, mit.DNA mitochondrial DNA

**
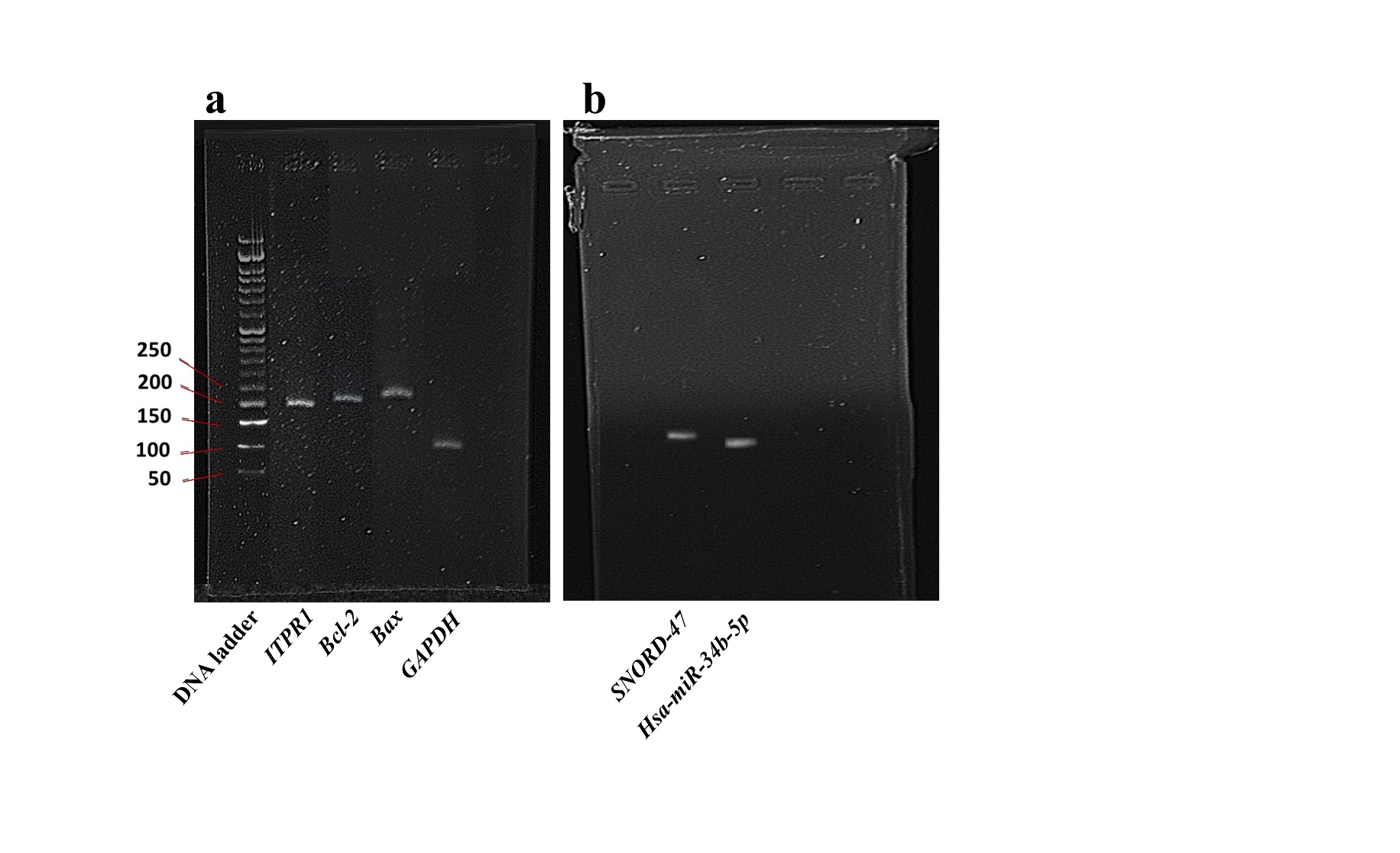
**

**
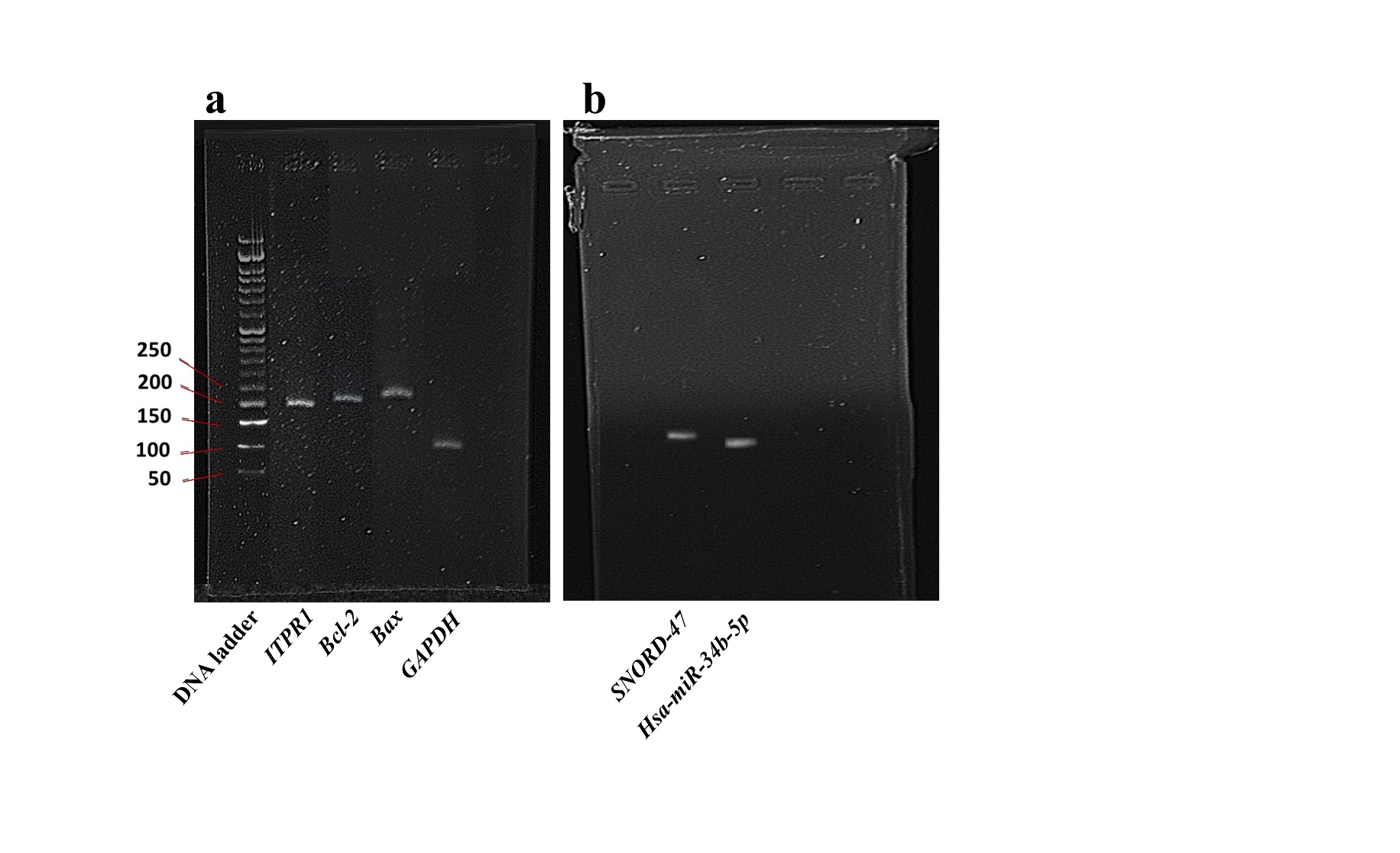
Supplementary Fig. S3: DNA gel electrophoresis for confirmation of gene amplification and primers specificity in RT-qPCR.** **a)** RT-qPCR products loaded on a 2% agarose gel stained with Ethidium Bromide. The first line accommodates a 50 bp ladder. Amplicon size of *ITPR1* =200 bp, *Bcl-2*= 232 bp, *Bax*= 244 bp, *GAPDH*=102 bp. **b)** RT-qPCR products loaded on a 4% agarose gel stained with Ethidium Bromide. The molecular size marker was not used because the amplicon size of *SNORD-47* and hsa-miR-34b-5p were unknown. Amplicon size of SNORD-47 and hsa-miR-34b-5p=Unknown.
